# Is there foul play in the leaf pocket? The metagenome of floating fern *Azolla* reveals endophytes that do not fix N_2_ but may denitrify

**DOI:** 10.1111/nph.14843

**Published:** 2017-10-30

**Authors:** Laura W. Dijkhuizen, Paul Brouwer, Henk Bolhuis, Gert‐Jan Reichart, Nils Koppers, Bruno Huettel, Anthony M. Bolger, Fay‐Wei Li, Shifeng Cheng, Xin Liu, Gane Ka‐Shu Wong, Kathleen Pryer, Andreas Weber, Andrea Bräutigam, Henriette Schluepmann

**Affiliations:** ^1^ Molecular Plant Physiology Department Utrecht University Padualaan 8 Utrecht 3584CH the Netherlands; ^2^ Department of Marine Microbiology and Biogeochemistry Netherlands Institute for Sea Research (NIOZ) Utrecht University Den Hoorn 1797SZ the Netherlands; ^3^ Department of Earth Sciences Utrecht University Utrecht 3508TA the Netherlands; ^4^ Department of Plant Biochemistry Cluster of Excellence on Plant Sciences (CEPLAS) Heinrich Heine University Düsseldorf 40225 Germany; ^5^ Max Planck Institute for Plant Breeding ADIS/DNA Core Facility Cologne 50829 Germany; ^6^ Institute of Botany and Molecular Genetics IBMG IRWTH Aachen University 52074 Aachen Germany; ^7^ Department of Biology Duke University Durham NC 27708 USA; ^8^ Boyce Thompson Institute for Plant Research Cornell University Ithaca NY 14853 USA; ^9^ Beijing Genomics Institute‐Shenzhen Shenzhen 518083 China; ^10^ Department of Biological Sciences University of Alberta Edmonton AB T6G 2E9 Canada

**Keywords:** ^15^N isotope, *Azolla*, denitrification, ditch water microbiome, metagenome, N_2_‐fixation, *Nostoc*, Rhizobiales

## Abstract

- Dinitrogen fixation by *Nostoc azollae* residing in specialized leaf pockets supports prolific growth of the floating fern *Azolla filiculoides*. To evaluate contributions by further microorganisms, the *A. filiculoides* microbiome and nitrogen metabolism in bacteria persistently associated with *Azolla* ferns were characterized.
- A metagenomic approach was taken complemented by detection of N_2_O released and nitrogen isotope determinations of fern biomass. Ribosomal RNA genes in sequenced DNA of natural ferns, their enriched leaf pockets and water filtrate from the surrounding ditch established that bacteria of *A. filiculoides* differed entirely from surrounding water and revealed species of the order Rhizobiales. Analyses of seven cultivated *Azolla* species confirmed persistent association with Rhizobiales.
- Two distinct nearly full‐length Rhizobiales genomes were identified in leaf‐pocket‐enriched samples from ditch grown *A*. *filiculoides*. Their annotation revealed genes for denitrification but not N_2_‐fixation. ^15^N_2_ incorporation was active in ferns with *N. azollae* but not in ferns without. N_2_O was not detectably released from surface‐sterilized ferns with the Rhizobiales.
- N_2_‐fixing *N. azollae*, we conclude, dominated the microbiome of *Azolla* ferns. The persistent but less abundant heterotrophic Rhizobiales bacteria possibly contributed to lowering O_2_ levels in leaf pockets but did not release detectable amounts of the strong greenhouse gas N_2_O.

## Introduction

Our growing global population is rapidly escalating the demand for nutritious food, requiring highly prolific and sustainable primary production. In tandem, the need for renewable feedstocks for the industry derived from primary production is also growing. To sustain future food and feedstock production, we need to explore novel crops that comply with limitations imposed by climate change, agrosystem inputs (e.g. water, fertilizers) and available arable land. Of particular concern is the ubiquitous requirement for nitrogen fertilizer in current agriculture that is tied to high input costs and negative climate consequences (Jensen *et al*., 2012). No crop plant is capable of fixing atmospheric dinitrogen (N_2_) autonomously. In leguminous crops such as soybean, plants recruit free‐living, nitrogen‐fixing bacteria in the order Rhizobiales from their environment anew each host generation (Vance, 2002), and it is within the legume's specialized root nodules that these symbiotic heterotrophic bacteria fix dinitrogen sufficient for host and bacteria. N_2_‐fixation requires large amounts of energy derived from the oxidation of plant sugars, which are a limiting factor. Under intensive agriculture, therefore, most leguminous crops are supplied with surplus nitrogen fertilizer to improve bean yields beyond 5 t ha^−1^ yr^−1^.

N_2_‐fixing cyanobacteria are known to form symbioses with other plants such as cycads, ferns and bryophytes (Adams *et al*., 2013). These symbiotic cyanobacteria can use light, as well as plant‐derived sugars, as an energy source to drive N_2_‐fixation. A single fern genus, *Azolla*, benefits from such a cyanobacterial symbiosis, and stands out for its prolific growth, resulting in high protein biomass without nitrogen fertilizer. For example, *Azolla filiculoides* produced 39 t ha^−1^ yr^−1^ dry weight (DW) biomass containing up to 25% protein (Becerra *et al*., 1990; Brouwer *et al*., 2014), whereas clover, *Trifolium pratense*, a high‐yielding forage legume that is commonly grown with low fertilizer applications (150 kg ha^−1^ yr^−1^), produced up to 15 t ha^−1^ yr^−1^ DW biomass containing similar protein amounts (Anglade *et al*., 2015). The cyanobacterial symbiont *Nostoc azollae* is key to the fern's remarkable productivity. With its genome highly degraded (containing 31.2% pseudogenes and over 600 transposable elements), *N. azollae* is unable to survive without its host (Ran *et al*., 2010), and its spores are vertically transmitted to the next fern generation via the fern megaspores (*sensu* Nagalingum *et al*., 2006) during sexual reproduction. The cyanobacteria reside within specialized leaf pockets of *Azolla*, where they form heterocysts with high frequency and utilize photosystem I to drive N_2_‐fixation. A colony of motile cyanobacteria typically resides at the meristematic tip of the branch where the leaf pockets of the young developing leaves are still open, allowing cyanobacteria to migrate inside (Perkins & Peters, 1993). Such a specialized environment that is attractive to cyanobacteria may also attract other bacteria. Electron micrographs of leaf and megasporangiate sori cross‐sections have revealed the presence of other bacteria in addition to the cyanobacteria (Carrapiço, 1991; Zheng *et al*., 2009). Immunohistochemical detection of nitrogenase using polyclonal antisera did not, however, unequivocally reveal nitrogenase in these bacteria (Braun‐Howland *et al*., 1988; Lindblad *et al*., 1991). Moreover, the number of species and taxonomy of the cyanobacteria associated with *Azolla* has been controversial (Pereira & Vasconcelos, 2014). The focus of our present study is to characterize the microbiome associated with *A. filiculoides*.

Plant–microbe interactions research has evolved rapidly over the last few decades (Turner *et al*., 2013). Most studies focus on microbial interactions between plant roots and the rhizosphere, whereas microbes in the phyllosphere, the above‐ground plant organs, have been less thoroughly examined (Peñuelas & Terradas, 2014). Symbiotic bacterial endophytes, nonpathogenic organisms that colonize intercellular spaces in plants, have been rediscovered with next‐generation sequencing approaches that permit *in situ* studies. Endophytes are ubiquitous and frequently found in plant species (Stone *et al*., 2000).

Although microbial species associated with a particular plant species can be numerous and diverse, a core microbiome can be identified, as illustrated for *Arabidopsis thaliana* (Lundberg *et al*., 2012). Microbiomes in the rhizosphere and phyllosphere are not the same, however, reflecting disparate niches in the different plant organs, each subject to its own developmental and environmental influences (Turner *et al*., 2013). These microbiome differences are not just opportunistic, but often convey beneficial properties to the host plant. Persistent mutualistic microbes have been shown in specific cases to increase the fitness of plants; for example, in legumes non‐nitrogen‐fixing rhizobia decrease grazing, thus eliminating the fitness costs of the mutualistic interaction (Simonsen & Stinchcombe, 2014). *Arthrobacter* species have been isolated repeatedly from *Azolla* and one strain was shown to produce the auxin IAA, possibly affecting fern development and growth (Forni *et al*., 1992); an *Agrobacterium* strain isolated from surface‐sterilized *A. filiculoides* assimilated ammonium, possibly sequestering the growth inhibitory nutrient when it accumulates in the leaf pockets in excess (Plazinski *et al*., 1990). The capabilities of the host and microbiome, the holobiont, should therefore be viewed as one unit reflected in the metagenome, evolving through myriad environmental constraints. This idea inspired the coining of *Azolla* as a ‘superorganism’ (Carrapiço, 2010).

Recently, shotgun sequencing of DNA extracted from microbial communities without PCR and subsequent metagenome assembly have become feasible, allowing for functional analyses of multiple genomes in addition to taxonomic assignments (Castelle *et al*., 2013; Wrighton *et al*., 2014). Assembly of short sequencing reads obtained from metagenome shotgun sequencing into long scaffolds, ideally representing near complete microbial genomes, however, remains elusive (Tyson *et al*., 2004; Charuvaka & Rangwala, 2011; Zependa Mendoza *et al*., 2015). Metagenome assembly quality is primarily influenced by the number and diversity of organisms present, as well as the length of reads. Improved assemblies with long scaffolds can be obtained by subcloning DNA into fosmids before sequencing or by using long‐read technologies such as those that were validated in studies of gut microbes (Mizuno *et al*., 2013; Leonard *et al*., 2014). The presence of an organism in an environmental sample may then be computed by recruiting short reads from the environmental sample onto the assembled genome of that particular organism, as was successfully demonstrated with phage genomes from the ocean or bacterial genomes from salt brines (Pašić *et al*., 2009; Mizuno *et al*., 2013).

The focus of the present study was to characterize the identity and function of microbes persistently associated with *A. filiculoides* using metagenomics shotgun sequencing of total DNA from samples collected in their natural environment, and also from cultured species of *Azolla*.

## Materials and Methods

### Plant materials

*A. filiculoides* Lam was obtained from the Galgenwaard ditch in Utrecht, the Netherlands. In addition, six *Azolla* species were obtained from the bio‐fertilizer germplasm collection at the International Rice Research Institute (IRRI) in the Philippines (Table 1; Watanabe, 1992).

**Table 1:** *Azolla* taxon sampling

Taxon	Origin
*Azolla filiculoides* Lam.	The Netherlands, Utrecht, Galgenwaard ditch, 52°4′35.73″N, 5°8′59.05″E
*Azolla filiculoides*‐Sterilized	Same as above; but surface sterilized and cultured on erythromycin to remove *Nostoc azollae* symbiont
*Azolla mexicana* Schltdl. & Cham. ex Kunze	aIRRI accession ME2001; originally from USA, California, Graylodge, collected by D. Rains in 1978
*Azolla microphylla* Kaulf.	aIRRI accession MI4021; originally from Ecuador, Galapagos, Santa Cruz Island; collected by T. Lumpkin in 1982
*Azolla nilotica* Mett.	aIRRI accession NI5001; originally from Sudan, Kosti; collected by T. Lumpkin in 1982
*Azolla caroliniana* Willd. (accession 1)	aIRRI accession CA3017; originally from Brazil, Rio Grande Sul; collected by I. Watanabe in 1987
*Azolla caroliniana* (accession 2)	aIRRI accession CA3004; originally from Uruguay, Treinta y tres; collected by D. Rains in 1982
*Azolla rubra* R. Br.	aIRRI accession RU6502; originally from Australia, Victoria, collected in 1985

a IRRI Bio‐Fertilizer Germplasm Collections (www.irri.org; Watanabe, 1992).

### Collection and processing of samples from the natural environment

Whole plants of *A. filiculoides*, its enriched leaf pocket contents and water filtrates from the surrounding water (13°C, pH 7.2) were collected as triplicate replicates from the Galgenwaard ditch in Utrecht (Table 1) on 28 October 2015. Plant and water replicates were carried from the collection site in separate containers and treated separately. Ferns were filtered using sieves of 4 mm mesh size to remove contaminating aquatic plants and animals, then washed by vortexing at full speed for 60 s in 0.5% Tween‐20, in batches of 5 g fresh weight (FW). For whole plant samples, one plant of 200 mg FW and two 3‐mm‐diameter glass beads were placed into tubes, snap frozen and then homogenized by a TissueLyser II (Qiagen). Leaf pocket‐enriched fractions were prepared from washed ferns as described by Orr & Haselkorn (1982). Ditch water (1 litre) from every replicate was passed through a 0.45 μm filter, and the biomass on the filter was then resuspended in 500 μl water and frozen (−80°C) until DNA extraction. DNA was extracted using the Mobio PowerLyzer PowerSoil kit (Qiagen), according to the manufacturer's protocol.

### Fern cultures and processing

Cultures of different *Azolla* species were obtained from the International Rice Research Institute (Philippines) except for *A. filiculoides* (Table 1; Watanabe, 1992). All *Azolla* species were grown on liquid medium without nitrogen and under long‐day light with a far‐red component as described by Brouwer *et al*. (2014), except where stated otherwise. To obtain sterilized cultures of *A. filiculoides*, explants (<1 mm^3^) of leaves from the ditch plants were surface‐sterilized using bleach at 1% available chlorine for 40 s, with four consecutive rinses in sterile water before cultivation on agar medium (0.6% (w/v) agarose, Duchefa, Haarlem, the Netherlands). *Azolla* without *N. azollae* (referred to here as *A. filiculoides‐*Sterilized) was first cultured on solid agar medium with 60 μg ml^−1^ erythromycin and 2 mM NH_4_NO_3_; the absence of *N. azollae* was verified using confocal microscopy and by quantitative PCR (see Fig. 4; Forni *et al*., 1991; Brouwer *et al*., 2014). Sterilized cultures were grown in enclosed glass containers with a stream of air (78 l h^−1^) pumped through 0.45 μm filters using aquarium pumps (SuperFish Air flow mini); *A. filiculoides‐*Sterilized were used for DNA sequencing, the ^15^N_2_‐fixation experiments and δ^15^N determinations of *Azolla* biomass. DNA extractions from cultured plants were, following enrichment for nuclei (Lutz *et al*., 2011), combined with the Genomic‐tip 100/G protocol (Qiagen) for the long read sequencing, or directly using the Genomic‐tip 100/G protocol for the short‐read Illumina sequencing.

### Sequencing library preparations and sequencing of DNA

Libraries for short‐read sequencing (in paired‐end mode) were made after shearing the DNA as per the recommended protocol (TruSeq Nano DNA Library Prep Kit, Illumina, Madison, WI, USA). For *Azolla* samples from the ditch, care was taken to shear the DNA to *c*. 800 bp (Covaris, Woburn, MA, USA) to improve Emirge assemblies. Sequencing was performed using the Illumina NextSeq500 desktop sequencer, yielding *c*. 3 Gb sequence information per replicate (Supporting Information Table S1). For cultured *Azolla* samples, libraries of 250, 500 and 800 bp were generated and sequenced at high coverage such that the data needed to be sub‐sampled to 10 and 30 million reads, for comparison with data obtained from ditch *Azolla*.

Libraries for PacBioRS II (Pacific Biosciences, Palo Alto, CA, USA) sequencing of the nuclear DNA from a single plant of *A. filiculoides*‐Sterilized (described under ‘Fern cultures and processing’) were generated after size separation with a cut‐off at 14 kb (Blue Pippin, Sage Science, Beverly, MA, USA) according to the PacBio RS II protocol and sequenced using P5‐C3 chemistry, reaching 57 times coverage of the 750 Mb genome.

### Taxonomic assignments based on small ribosomal RNA (sRNA) sequences

Short‐read sequences were sorted according to biological replicates and paired‐end reads were trimmed using Trimmomatic (parameters ‘LEADING:5 TRAILING:5 SLIDINGWINDOW:4:15 MINLEN:36’; Bolger *et al*., 2014). All reads passing quality control (QC) were processed in parallel by RiboTagger, which directly assigns taxonomy from variable regions of rRNA genes found in single reads using a subset of the Silva database containing the V4–V7 variable regions as reference (Tange, 2011; Xie *et al*., 2016). Nearly whole‐length rRNA genes were assembled with Emirge using standard parameters over 120 iterations (Miller *et al*., 2011). Classification of assembled rRNA genes was performed by Mothur, using the Silva nonredundant v119 reference database (Schloss *et al*., 2009; Quast *et al*., 2013). In addition to processing samples as individual replicates (P_1__to__3_, L_1__to__3_, W_1__to__3_), reads from the three biological replicates of whole plant, leaf juice or water were pooled (P, L and W, respectively) before analyses with either RiboTagger or Emirge. This was done to evaluate the sensitivity of the taxon detection using either Emirge or RiboTagger with three times more reads.

### Genome assemblies with long reads

Long reads (PacBioRS II) from DNA of *A. filiculoides*‐Sterilized were read‐corrected and then assembled into scaffolds by both the Celera and Falcon assembler pipelines, yielding two preliminary genome assemblies (Myers *et al*., 2000; https://github.com/PacificBiosciences/FALCON; Koren *et al*., 2012). Bacterial scaffolds in the genome assemblies were identified by RNAmmer (Lagesen *et al*., 2007). Bacterial scaffolds with a minimum length of 0.1 Mb were extracted and assigned taxonomy based on the 16S rRNA genes in Mothur using the Silva database (Table 2). Once identified, the scaffolds were submitted to Rast (Overbeek *et al*., 2014) for annotation, which scored the nearest neighbor.

**Table 2:** Bacterial scaffolds found in genome assemblies of *Azolla filiculoides*‐Sterilized identified by RNAmmer and annotated by Rast

Assembly method^a^	Genus (Mothur/Silva)^b^	Length (bp)^c^	Features after Rast annotation (missing genes)^d^	Denitrifying (N‐metabolism genes)^e^	Closest relative (Rast)^f^
Celera	Unknown	7478			
Celera	*Microbacterium*	23 491			
Celera	*Hyphomicrobium*	16 162			
Celera	*Shinella*	283 870	259	No (0)	*Sinorhizobium meliloti*
Celera	*Shinella*	4962 292	4811 (36)	Yes (27)	*Sinorhizobium meliloti*
Celera	*Ralstonia*	1425 495	1312 (18)	Yes (21)	*Ralstonia pichettii*
Celera	*Ralstonia*	2321 690	2200 (15)	No	*Ralstonia pichettii*
Celera	*Rhizobium*	28 900	362	Yes (4)	
Celera	*Rhizobium*	807 886	758	Yes (6)	*Rhizobium leguminosarium*
Celera	*Rhizobium*	1061 533	1853	Yes (5)	*Rhizobium leguminosarium*
Celera	*Rhizobium*	3220 799	3178 (6)	No (9)	*Agrobacterium tumefaciens*
Celera	*Hydrocarboniphaga*	2071 427	1856	No	*Hydrocarboniphaga effusa*
Celera	*Hydrocarboniphaga*	3085 094	2672 (164)	No	*Hydrocarboniphaga effusa*
Falcon	*Rhizobium*	413 8029	6897 (31)	Yes (26)	*Sinorhizobium meliloti*

a PacBioRSII reads were read‐corrected then assembled using either the Celera or the Falcon pipelines. The *Sinorhizobium*‐like scaffold was assembled by both pipelines yielding 4.906 Mb and 4.138 Mb scaffolds, respectively, for Celera and Falcon. These sequences were largely identical but Rast annotation of the N‐metabolism genes differed by one gene (Overbeek *et al*., 2014).

b RNAmmer detected rRNA genes in the scaffolds and taxonomy was based on the rRNA gene sequences with Mothur using the Silva database.

c Length of the scaffolds in base pairs.

d Number of features computed by Rast annotation including the number of missing genes in parentheses.

e Presence of genes from the denitrifying pathway with the total number of nitrogen metabolism genes in the scaffold in parentheses. Small scaffolds from singleton genera were omitted.

f The closest relative as computed by Rast.

### Recruitment analyses

Short‐read sequences were mapped to reference scaffolds and genomes with Bowtie2 (v.2.2.6; options: –very‐sensitive (‐D20‐R3‐N0‐L20‐iS1,0.50); Langmead & Salzberg, 2012). If applicable, fragmented genomes were converted to one sequential sequence for the purpose of visualization. Bowtie output was parsed with a custom script to extract position and the common bases in the alignment (identity score). In a custom R script, aligned reads were binned (normalized for 0.05 Mb and 1% identity) and read count per bin was log_10_‐transformed (Wickham, 2011; R Core Team, 2013; Dowle *et al*., 2014; Carr *et al*., 2015).

### Data deposition

The sequences reported in this paper have been deposited in the ENA database with the study accession number PRJEB19522; the data are separated into three categories: Illumina paired end NextSeq500 sequences (2 × 150 bases (b)) from the environmental samples, Illumina paired end NextSeq500 sequences (2 × 150 b) and short‐read sequences sampled at 30 M reads from each of the different species and bacterial scaffolds (including PacBioRSII‐corrected reads).

### ^15^N_2_ fixation, δ^15^N determinations and N_2_O release

Surface‐sterilized ferns (100 mg FW) were placed in enclosed bottles with 43 ml of sterile medium and a residual air space of 262 ml. To determine N_2_ fixation after 2 h, ^15^N_2_ (15 ml) was added at 14 h using air‐tight syringes whilst overpressure was removed using a release needle; the bottles were then incubated for 2 h under growth conditions as in Brouwer *et al*. (2014). To determine N_2_ fixation after 24 h, ^15^N_2_ (5 ml) was added as well as CO_2_ (5 ml). After the incubation with ^15^N_2_, samples were snap frozen in liquid nitrogen, freeze‐dried and homogenized before analysis of the dry weights, N content and isotope abundance determinations. In both the 2 and the 24 h incubation experiments, ^15^N_2_ provided from Sigma was washed with acid to remove ammonia. In the 24 h incubation experiment the gas was washed in addition with a base to remove NOx.

Total N content and stable nitrogen isotopes (δ^15^N) were analyzed on a ThermoScience Delta Plus isotope ratio mass spectrometer connected on‐line to a Carlo Erba Instruments Flash 1112 elemental analyzer. We assumed no isotope discrimination during the fixation process and therefore rates of fixation calculated may be underestimated.

Ferns used for N_2_O measurements included *A. filiculoides* cultured in the laboratory (nonsterile), and surface‐sterilized ferns with and without *N. azollae* (*A. filiculoides*‐Sterilized) grown under sterile conditions. For experiments with nonsterile material, 10 g FW fern was used with 200 ml air headspace. For experiments including sterile materials, 100 mg FW fern was used with 15 ml micro‐aerobic (10% (v/v) O_2_) head space. Gas samples of 6 ml were separated on a Hayesep Q column by GC (Hewlett Packard Agilent Technologies) and gases were detected with an electron capture detector (ECD 63 Ni).

## Results

### *Azolla filiculoides* sustains a unique microbiome

**Figure 1:**
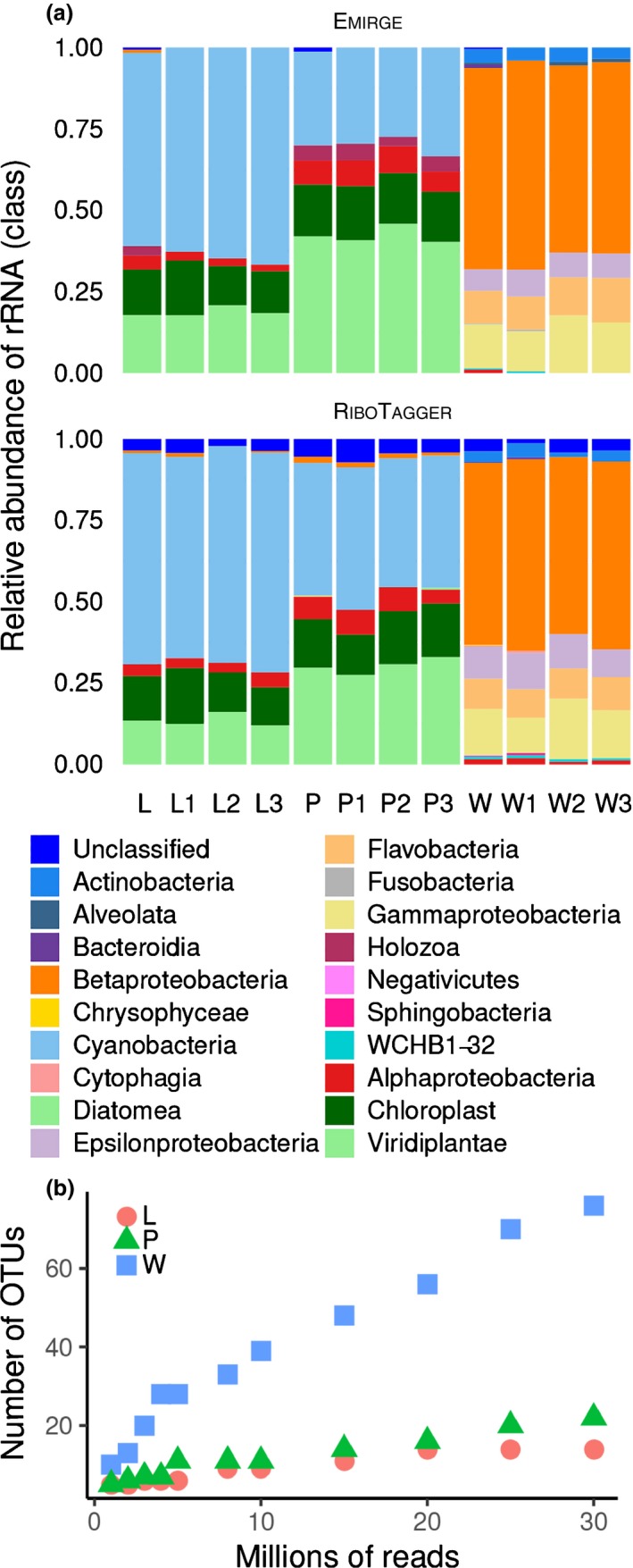
Taxonomic diversity revealed in DNA isolated from ditch samples of *Azolla filiculoides*. Sequence data originating from leaf pocket‐enriched samples (L), whole plants (P) and surrounding ditch water (W) were processed as separate biological triplicates, and as a pool thereof. (a) Relative abundance of bacterial classes derived from rRNA assemblies with Emirge combined with taxonomic assignments with Mothur (Emirge) or from RiboTagger analyses of reads with rRNA variable regions (RiboTagger). (b) RiboTagger operational taxonomic units (OTU) count with increased reads from pooled samples of leaf pocket‐enriched samples (L), whole plants (P) and surrounding ditch water (W).

The Dutch ditch plants of *A. filiculoides*, together with samples of their *in situ* ditch water, were sampled and processed for sequencing independently in three biological replicates (*i* = 1–3) of the following types: whole plant (P_i_), enriched leaf pocket contents (L_i_) and surrounding water (W_i_), containing 8.42–11.99 M reads averaging 147 b (Table S1). Taxonomic groups present in samples were computed either by rRNA assembly with Emirge or by analysis of reads containing 16S rRNA variable regions with RiboTagger, using the Silva rRNA reference database (Miller *et al*., 2011; Quast *et al*., 2013; Xie *et al*., 2016). The distribution of taxonomic classes or orders over replicate samples was similar for both methods and very similar among biological replicates (Fig. 1a). RiboTagger taxonomic assignments were not influenced by the number of reads sampled (10 or 30 M) since it computed an identical set of classes or orders in replicates with 10 M reads compared to when the three replicates were pooled to submit 30 M reads for analysis. When assembling rRNA genes with Emirge, however, pooling replicates before Emirge assembly occasionally yielded more taxonomic assignments, probably because assemblies were dependent on read coverage (Figs S1, S2).

Ditch water surrounding *A. filiculoides* was more diverse in its microbial community composition than were the plant‐related samples: the mean Shannon diversity of RiboTagger‐assigned microbial taxonomy was 3.11 ± 0.16 (SD) for water samples, compared to 1.47 ± 0.07 and 1.11 ± 0.08 for whole plant and leaf samples, respectively. The community richness was also higher in the ditch water samples than in plant‐related samples. Rarefaction analysis showed saturation of the plant‐associated microbiome with sampling size, but not for the ditch water (Fig. 1b). Over half of the taxa found in water samples were identified as class Betaproteobacteria, with the orders Burkholderiales, Rhodocyclales and Methylococcales being the most abundant (Fig. 1a). Overlap between *Azolla*‐associated and water samples was zero at order level and minimal at class level.

### *Nostoc azollae* is the most abundant endophyte of *Azolla filiculoides*

Taxonomic identification revealed a conserved and plant‐specific microbial community associated with *A. filiculoides* (Fig. 1a: L, P). Most rRNA hits were assigned to either fern chloroplasts, Viridiplantae nuclei or cyanobacteria. Cyanobacteria‐derived rRNA sequences were more abundant in the enriched leaf pocket contents than in the whole plant samples. Fern mitochondrial rRNA was absent from the database and instead assigned to the order Ricketsiales (class Alphaproteobacteria) that was systematically present in all whole plant *Azolla* samples, yet less abundant in leaf pocket‐enriched samples. Cyanobacteria‐related sequences were the most abundant in all fern samples, making up *c*. 60–75% and 45% of the rRNA hits in L and P samples, respectively (Fig. 1a: L, P). The accuracy of assembled 16S rRNA genes was confirmed by aligning the rRNA assemblies assigned to cyanobacteria to the *N. azollae* 16S rRNA gene (NCBI reference sequence: NR_074259.1): multiple sequence alignment with ClustalW revealed over 99.5% similarity over the full length of the alignment. The results therefore confirmed that *N. azollae* is the primary symbiont of *A. filiculoides*.

### Rhizobiales are constitutive members of the microbiome in natural and cultivated *Azolla* species

**Figure 2:**
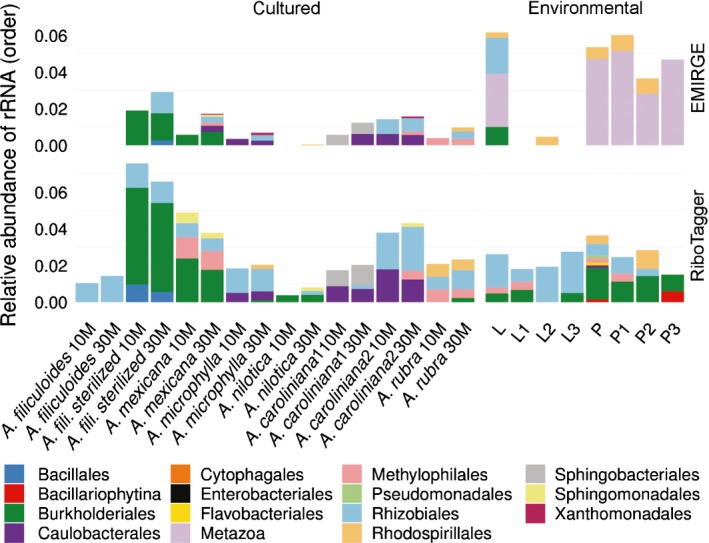
Relative abundance of orders within cultured species of *Azolla* (Table 1) and ditch samples of *Azolla filiculoides* (natural and sterilized). Taxonomy was assigned to rRNA fragments found in single reads by RiboTagger (RiboTagger) and to rRNA genes assembled with Emirge by Mothur (Emirge). Unclassified orders or those originating from *Viridiplantae* nuclei, fern plastids and cyanobacteria are not shown. Environmental sequencing data originated from *A. filiculoides* leaf pocket‐enriched samples (L) and whole plants (P) in biological triplicates. Sequence reads from cultured ferns were processed as subsets of 10 M and 30 M reads.

To help reveal microorganisms associated at low abundance with *A. filiculoides* from the ditch, we removed rRNA hits derived from chloroplasts, Viridiplantae nuclei, mitochondria, cyanobacteria and unclassified sequences (Fig. 2, Environmental). RiboTagger found more operational taxonomic units (OTUs) in nearly all samples than did Emirge. Only Emirge, however, found Metazoa 18S rRNA in all *Azolla* plant (P) and one leaf pocket‐enriched (L) samples. These rRNA genes all mapped to *Stenopelmus rufinasus*, a weevil specialized in feeding on *Azolla* (Hill, 1998). All five assembled Metazoa rRNA genes and GenBank reference FJ867794.1 were trimmed to corresponding lengths and aligned: 98.2% of the 1200 bp multiple sequence alignment was identical. Detection of the weevil and the perfect assembly of the *N. azollae* rRNA confirmed the accuracy of Emirge assemblies and subsequent taxonomic assignments by Mothur. The bacterial orders Rhizobiales and Burkholderdiales were found enriched in L samples by both methods at 2% and 1% abundance, respectively, and in all but one L sample by RiboTagger (Fig. 2, Environmental).

For the cultured *Azolla* species, short‐read sequencing data obtained from seven different species were also analyzed using Emirge and RiboTagger (Table 1). Cultured ferns included *A. filiculoides* originating from the same ditch as the environmental sample but cultured for 2 yr so as to be devoid of *N. azollae* (=*A. filiculoides*‐Sterilized). The most abundant taxonomic assignments from DNA of cultured *Azolla* species were Viridiplantae nuclei, chloroplast and cyanobacteria (Fig. S1); these were removed to reveal taxa present at a lower abundance (Fig. 2, Cultured). Members of Burkholderiales, present in ditch samples of *A. filiculoides*, were infrequently observed in cultured *Azolla* species. However, they were particularly prominent in *A. filiculoides‐*Sterilized. Similarly, Caulobacteriales were infrequently observed in cultured *Azolla*. By contrast, Rhizobiales were observed in all cultured and environmental *Azolla* samples, including those devoid of *N. azollae* (Fig. S2, *A. filiculoides‐*Sterilized). *Azolla* accessions from IRRI had been cultured for many years (Table 1), raising the likelihood that their microbiomes were considerably altered from when first collected in their natural environment. The persistent occurrence of Rhizobiales in environmental, cultured and sterilized ferns, however, suggested that these bacteria are closely associated with the fern and possibly have an added ecological function in the *Azolla–Nostoc* symbiosis. Detection of the rRNA genes from Rhizobiales in DNA from *A. filiculoides*‐Sterilized further indicated that the long‐read nuclear genome assembly from this plant probably contained scaffolds of persistent bacterial endophytes.

### Near full‐length genomes of two novel *Rhizobiales* species in assemblies of the *Azolla filiculoides* genome are present in all *Azolla* species

The Falcon and Celera assemblies from the *A. filiculoides*‐Sterilized were scanned for bacterial scaffolds (presence of 16S rRNA) with RNAmmer; scaffold taxonomy was then assigned using Mothur if they were longer than 0.1 Mb (Table 2). Both assemblies reproducibly yielded scaffolds from the genera *Shinella* and *Rhizobium* (Rhizobiales).

**Figure 3:**
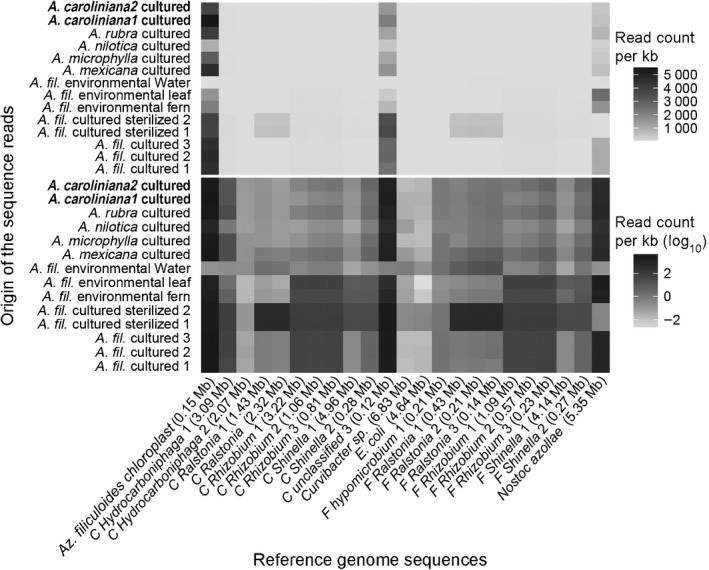
Recruitment summary on bacterial scaffolds obtained by Celera or Falcon assemblies of the *Azolla filiculoides* genome. Short reads from cultured *Azolla* species and environmental samples, *A. filiculoides* leaf pocket‐enriched and whole plant (L, P) and water control (W), were recruited onto assembly scaffolds including the *A. filiculoides* chloroplast as well as on to *Escherichia coli* (GCA_000005845.2_ASM584v2) and *Nostoc azollae* (NC_014248.1) reference genomes. Bacterial scaffolds were from *A. filiculoides* genome assemblies computed with either Celera (C) or Falcon (F) pipelines; length of the scaffolds is in megabases (Mb). Read counts were normalized per kilobase with color coding in linear scale (top panel) illustrating the dominance of DNA from chloroplast and *N. azollae*. Normalized read counts were further scaled logarithmically (bottom panel) to reveal differences between the negative control *E. coli* and presence calls for scaffolds belonging to the bacterial genera *Hydrocarboniphaga*,* Rhizobium* and *Shinella*.

To differentiate true symbiotic partners from contaminations due to culture treatments or DNA extractions, short reads of all cultured species and environmental samples were mapped to the extracted scaffolds: only hits with an identity over 97% were counted and hit frequency was normalized for scaffold length, thus generating a heat map (Fig. 3). Scaffolds assigned to *Ralstonia* (Burkholderiales) were most abundant in samples of *A. filiculoides‐*Sterilized, but absent in other species. Three other bacterial genera present in multiple *Azolla* species stood out with substantial counts: *Hydrocarboniphaga* (Nevsikiales), and *Shinella* and *Rhizobium* (Rhizobiales). The three *Rhizobium* and two *Shinella* scaffolds had the same relative frequencies in each sample, indicating that they each originated from one species of *Rhizobium* and *Shinella*, respectively. Scaffolds from the Rhizobiales were on average more frequently mapped by reads from the leaf pocket‐enriched (L) samples than from whole plants (P); enrichment locates the bacteria from the *Rhizobium* genome in the leaf pockets (Fig. S3).

**Figure 4:**
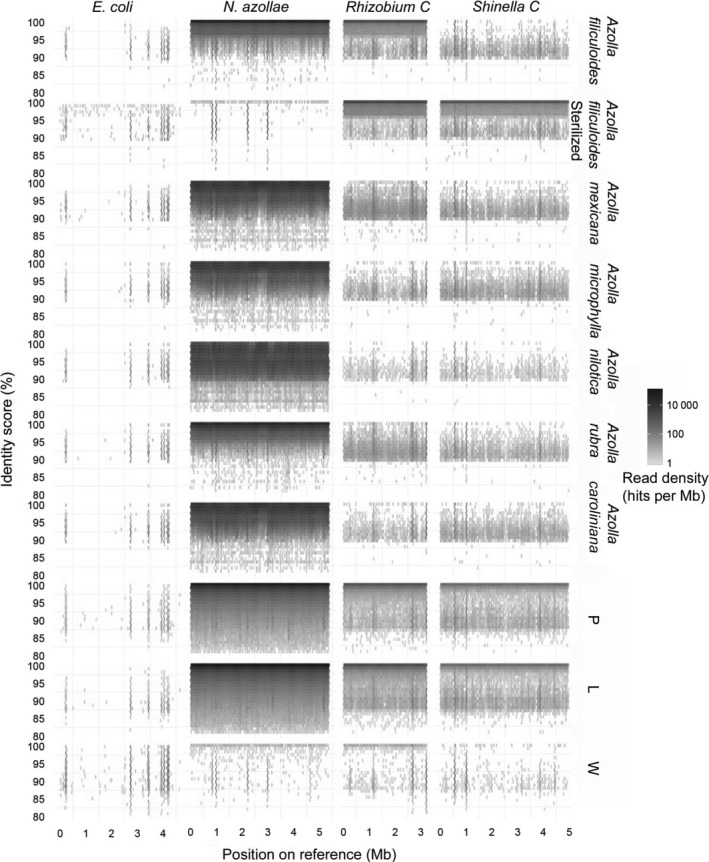
Recruitment analysis using short reads from cultured and environmental *Azolla* and water samples onto reference genomes of *Nostoc azollae* (GenBank CP002059.1), *Escherichia coli* (GCA_000005845.2_ASM584v2), the *Shinella* scaffold and two *Sinorhizobium meliloti* genomes (AL591688.1 and AKZZ01000000, respectively). Reads were from DNA of cultured ferns or from the ditch samples as in Figs 1, 2, 3 (see also Table 1). All reads were mapped with Bowtie (options: –very‐sensitive) and identity scores were calculated with a custom script (see the Materials and Methods section). Reads were binned according to identity score and position on the respective genome, then counted per 50 kb for normalization, and counts were log_10_‐transformed. L, leaf pocket‐enriched samples; P, whole plants; W, surrounding ditch water.

To evaluate their representation in the data over the full length of their genomes, short reads of all cultured and environmental samples were mapped to the longest scaffolds of these bacterial genera (Fig. 4). High identity reads (100%) mapped with high frequency to the *N. azollae* genome, revealing that the published *N. azollae* genome is the same species as that found in *A. filiculoides* from the Dutch ditch. The absence of reads from the *A. filiculoides‐*Sterilized samples mapping to *N. azollae* confirmed that these plants were devoid of cyanobacteria. In the genomes that were absent from these samples, sporadic loci still mapping reads with high identity were localized at highly conserved genes such as rRNA. By contrast, the 3.2 Mb *Rhizobium* and 4.9 Mb *Shinella* scaffolds were represented over the full length of the scaffolds in all fern samples. High identity reads were more abundant in *A. filiculoides* environmental and cultured samples compared to other *Azolla* species; nevertheless, these scaffolds were mapped with over 90% identity over their full length in all *Azolla* species. The *Hydrocarboniphaga* scaffold was only highly represented in fern samples in an area confined to the end of the scaffold; this scaffold therefore was probably an artefact of assembly fused at its end to *A. filiculoides* genomic DNA (Fig. S4).

### The *Rhizobiales* endophytes of *Azolla filiculoides* contain denitrification enzymes

To explore possible functions of bacteria from the *Azolla* microbiomes identified during our recruitment analysis, the combined *Rhizobium* and combined *Shinella* scaffolds were submitted for annotation to Rast (Aziz *et al*., 2008; Overbeek *et al*., 2014), which computed that the most similar organisms were, respectively, *Agrobacterium tumefaciens* and *Sinorhizobium meliloti* (Rhizobiales).

To evaluate the relatedness of our *Sinorhizobium*‐like genome with the two known *S. meliloti* genomes (GenBank AL591688.1 and AKZZ01000000), we mapped reads from environmental samples and *A. filiculoides*‐Sterilized to these genomes (Fig. S4). Whilst the *Sinorhizobium*‐like genome was well represented in all *Azolla* samples, reads of all ditch and cultured fern samples mapped less efficiently to both known *S. meliloti* genomes. The *Sinorhizobium*‐like endophyte was thus determined to be a distinct species from *S. meliloti*. Similarly, the *Agrobacterium*‐like endophyte persistently detected in all *Azolla* ferns (Fig. 4) was distinct from known *A. tumefaciens* strains.

**Figure 5:**
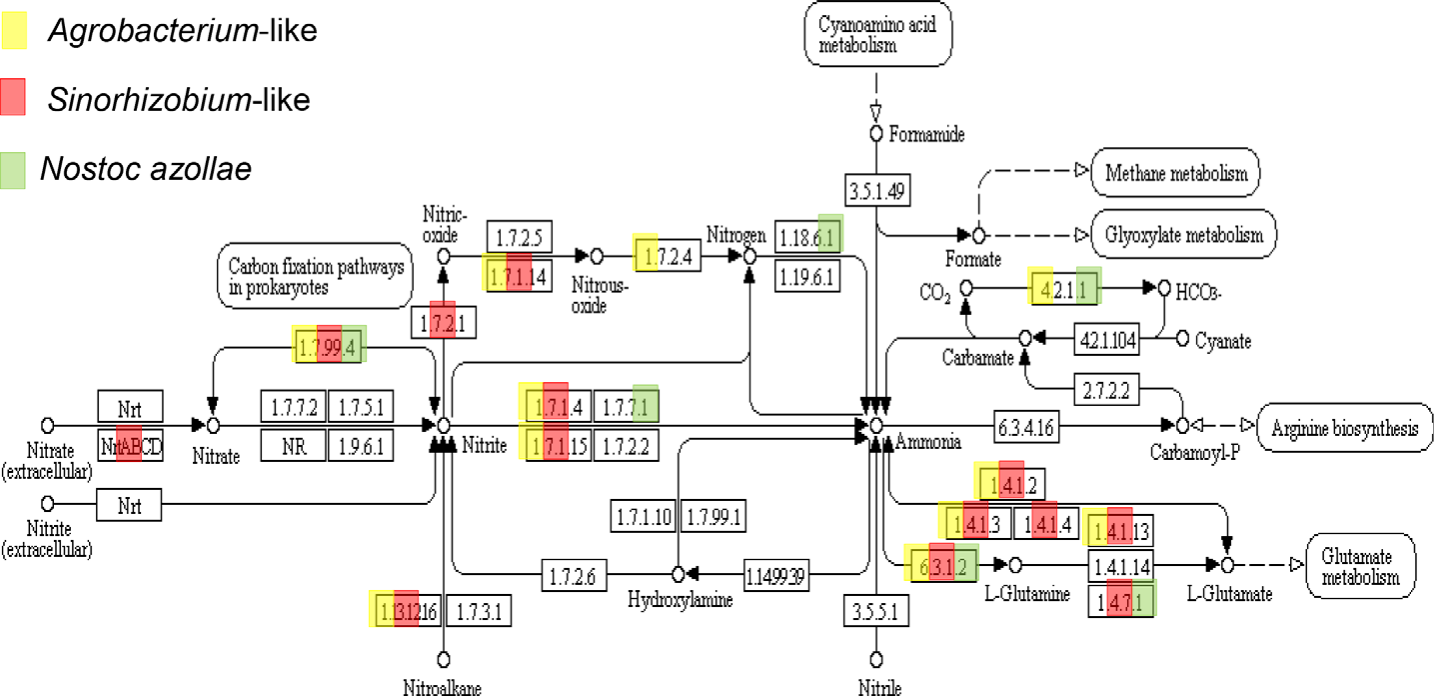
Nitrogen metabolism pathway comparing merged *Agrobacterium*‐like and *Sinorhizobium‐*like genomes and *Nostoc azollae*. The KEGG database was used to retrieve proteins from the closest relative, which was manually annotated (Kanehisa *et al*., 2010), and the proteins were then aligned via Blast to the merged scaffolds using the Rast/Seed viewer tool (Overbeek *et al*., 2014). The KEGG‐map of the nitrogen metabolism pathway was used to color‐in proteins detected in the merged scaffolds named after the closest relative computed by Rast, or in the *N. azollae* genome using the KEGG/NCBI annotation: *Agrobacterium*‐like (yellow), *Sinorhizobium*‐like (red) and *N. azollae* (green).

Analyses of N‐cycle coding genes revealed that both Rhizobiales genomes were lacking the N_2_‐fixing nitrogenase but instead encoded proteins from the denitrifying pathway (Fig. 5; Table S2). The *Sinorhizobium*‐like genome contained intact nitrite reductase, nitric oxide reductase and their accessory proteins (Figs S5, S6). The *Agrobacterium*‐like genome did not contain nitrite reductase but contained nitric oxide reductase and nitrous oxide reductase features. Closer inspection of the locus and protein alignment, however, revealed insertions of mobile elements in key genes of the nor and nos operons (Figs S7, S8). Rhizobiales endophytes hosted by *Azolla* ferns therefore did not contribute to N_2_‐fixation but may have released N_2_O and possibly also N_2_.

### *Azolla filiculoides* lacking cyanobacteria, but with the *Rhizobiales* present, neither fix nitrogen nor release detectable amounts of N_2_O

**Figure 6:**
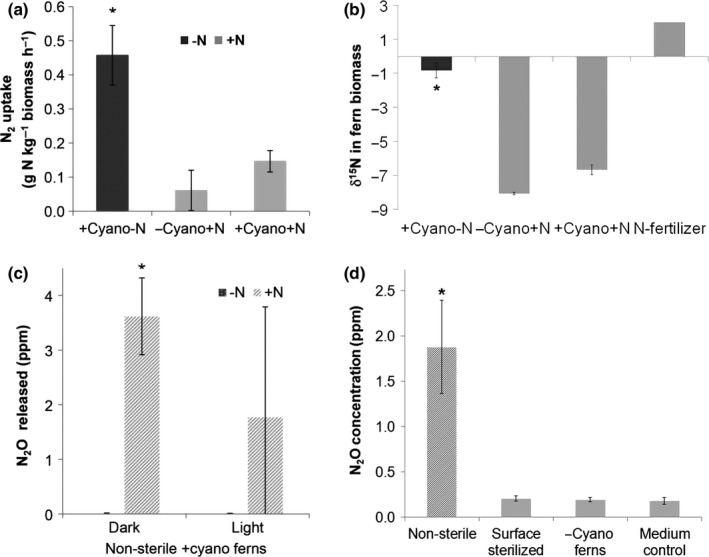
Is nitrogen (N) cycling inside *Azolla filiculoides*? *A. filiculoides‐*Sterilized were cultured in sterile medium with 2 mM NH_4_ NO_3_ (–cyano+N) and compared to surface‐sterilized *A. filiculoides* (with *Nostoc azollae*) growing in sterile medium without NH_4_ NO_3_ fertilizer (+cyano‐N) and with 2 mM NH_4_ NO_3_ (+cyano+N). (a) Midday uptake of ^15^N_2_ after 2 h exposure to ^15^N_2_‐enriched air. (b) δ^15^N in the biomass of the clonal *A. filiculoides* as in (a) but without ^15^N_2_‐enriched air. (c) Nonsterile *Azolla* containing cyanobacteria (+cyano) were used to detect N_2_O released after 6 h comparing light and dark, and +/−N medium. (d) N_2_O concentration after 6 h of incubation in the dark at the end of the night in micro‐aerobic (*c*. 10% (v/v) O_2_) air‐space from *A. filiculoides* as in (a), and nonsterile *Azolla* containing cyanobacteria growing on +N medium and from +N medium. Means ± SD for *n *=* *3 are shown. *, Significant difference with −cyano ferns (a), with −N medium (c) and with medium control (d); *P *<* *0.05, Student's *t*‐test.

Nitrogen‐fixation in surface‐sterilized *A. filiculoides* with and without *N. azollae* (*A. filiculoides*‐Sterilized) that were infected with the Rhizobiales endophytes was examined by supplying ^15^N_2_ at mid‐day for 2 h (Fig. 6a), when both CO_2_ and N_2_ fixation peak (Brouwer *et al*., 2014). ^15^N_2_‐fixation was not significant in *A. filiculoides*‐Sterilized (Fig. 6a, –Cynao+N). Whilst N_2_‐fixation was inhibited by N‐fertilizer in the medium required to sustain growth of *A. filiculoides*‐Sterilized (Fig. 6a, compare +Cyano‐N with +Cyano+N), *A. filiculoides* with *N. azollae* fixed significant amounts of nitrogen even after 2 h (Fig. 6a, +Cyano+N). When examining nitrogen fixation after one diel cycle of 24 h incubation with ^15^N_2_, δ^15^N of the biomass was still not significantly increased in *A. filiculoides*‐Sterilized compared to the boiled control whilst it reached on average 362 in ferns with cyanobacteria (Fig. S9). Endophytic Rhizobiales in *A. filiculoides*‐Sterilized therefore did not fix N_2_. This result was consistent with the absence of the N_2_‐fixing pathway in our Rhizobiales genomes (Fig. 5). In air without ^15^N_2_ added, biomass δ^15^N of the ferns with cyanobacteria in the absence of N‐fertilizer was much higher than with fertilizer (Fig. 6b, +cyano‐N vs +cyano+N), consistent with inhibition of N_2_‐fixation on media with 2 mM NH_4_NO_3_ in Fig. 6(a). The most negative δ^15^N in *A. filiculoides*‐Sterilized confirmed the absence of N_2_‐fixation in these ferns (Fig. 6a).

N_2_O release was robustly detected when assayed after 6 h in darkness using nonsterile *Azolla* on medium with 2 mM NH_4_NO_3_, but not on medium without nitrogen fertilizer (Fig. 6c), even after much longer than 6 h incubation (data not shown). Dependence of N_2_O release on medium with N suggested that if any N_2_O was synthesized in the leaf pockets it would be efficiently converted into N_2_. In contrast to nonsterile *A. filiculoides*, N_2_O release was not detected when *A. filiculoides*‐Sterilized were grown used on medium with N‐fertilizer after 6 h of darkness at the end of the night and in a micro‐oxic air space (Fig. 6d). N_2_O release from nonsterile *A. filiculoides* therefore probably originated from bacteria loosely associated with the fern surface, not from the endophytes. The results were consistent with the low abundance of the denitrifying Rhizobiales endophytes (Fig. 2).

## Discussion

### *Nostoc azollae* is abundant and the only cyanobacterium that fixes N_2_ in *Azolla filiculoides*

*N. azollae* in *A. filiculoides* from the present study and the published strain from Stockholm (Ran *et al*., 2010) were the same species based on the above 97% identity of their rRNA. Our analyses in Fig. 1 showed enrichment of *N. azollae* rRNA in the leaf juice and did not detect any rRNA from another cyanobacterial species, suggesting that in the Utrecht ferns, *N. azollae* was the only abundant cyanobacterium in the leaf pockets; Figs 6 and S9 further demonstrated that *N. azollae* was responsible for N_2_‐fixation in the ferns. The large number of reads that mapped to the *N. azollae* genome with < 100% identity in the recruitment analyses (Fig. 4) were probably explained by natural variation in bacterial populations and activity of insertion elements in *N. azollae* (Vigil‐Stenman *et al*., 2015). Previous reports suggesting that several species of cyanobacteria may inhabit the leaf pockets (Gebhardt & Nierzwicki‐Bauer, 1991) may have described very low abundance cyanobacteria not detected by our analyses, which revealed bacteria with a relative rRNA abundance at relative detection limit of 0.2%. Our analyses confirmed the presence of less abundant Gram‐negative eubacteria in leaf pockets of *A. filiculoides*, in particular that of an *Agrobacterium* strain (Plazinski *et al*., 1990).

### Two novel candidate bacterial species from the Rhizobiales are persistent endophytes of all *Azolla* species

Our data support that *Azolla* has control over the bacterial community assembly within its closed leaf pockets. First, the bacterial community of the surrounding ditch water was dominated by Proteobacteria, which are typically found in Dutch ditches (El‐Chakhtoura *et al*., 2015), and had no overlap with taxa within the *Azolla* leaf pocket. Second, different *Azolla* species cultured under the same conditions housed reproducibly different assemblages of microbial endophytes (Fig. 2, Cultured). Third, Rhizobiales endophyte genome scaffolds were recovered from sequencing nuclear preparations of *A. filiculoides*‐Sterilized; this *Azolla* strain had been grown on erythromycin then cultured in sterile conditions for over 2 yr (Fig. 4). In accordance, Arabidopsis leaf endophytes were shown to depend on the plant genotype, thus demonstrating that the plant host controls the assembly of endophytic bacterial communities (Horton *et al*., 2014); gene loci that influenced the bacterial communities, for example, encoded regulators of viral reproduction, pectin metabolism and trichome development. The *Azolla* control over the leaf pocket bacterial community may also depend on the presence of cyanobacteria, since Burkholderiales were more abundant in *A. filiculoides‐*Sterilized (Fig. 2a). The more general lesson learnt was that bacterial scaffolds in genome assemblies deserve attention as they may represent persistent endophytic bacteria.

Rhizobiales bacteria were found in all species of *Azolla* examined, despite the low proportion of reads with 16S rRNA sequences when sequencing all DNA extracted from the ferns or leaf juice compared to when sequencing PCR‐amplified rRNA genes. The difference in the 10 and 30 M read‐based taxonomy assignments using Emirge/Mothur in Fig. 2 and no saturation in Fig. 1(b) attest to this limitation. Rhizobiales were also reproducibly detected in the leaves of several species from the carnivorous angiosperm *Genlisea* using the meta‐transcriptomics approach, which will yield proportionally more rRNA sequences because of the high accumulation of rRNA in RNA extracts (Cao *et al*., 2015). The long‐read assembly of bacterial scaffolds combined with recruitment analyses, however, allowed a very high resolution of the taxonomic assignments in the present study. With Rast, closest relatives were computed scoring homologies of gene candidates predicted by Glimmer3 with a set of universal proteins and 200 unduplicated proteins (Overbeek *et al*., 2014). Recruitment analyses further refined this and showed that the *Sinorhizobium*‐like endophytes in *A. filiculoides* were not the *S. meliloti* species known (Fig. S4), yet were more than 90% identical over the genome length when comparing the differing *Azolla* species (Fig. 4). Furthermore, calculating read counts per kilobase in Fig. S3 quantified enrichment of Rhizobiales in leaf‐pocket content compared to plant samples, thus locating *Agrobacterium*‐like bacteria preferentially in the leaf pockets. Unlike the cyanobacteria in the leaf pockets, the Rhizobiales endophytes did not fix N_2_ and were present in much lower abundance, as judged from the recruitment analyses.

### A possible role for denitrifying *Rhizobiales* of the *Azolla* metagenome

Persistent Rhizobiales endophytes with denitrifying pathways suggested there may be some wasted cycling of the fixed nitrogen that is not likely to be of direct benefit to *Azolla* (Fig. 5). In the absence of N fertilizer *Azolla* will thrive entirely on N_2_ fixed by *N. azollae*; this explained the low δ^15^N of the fern biomass grown without N fertilizer compared to legume biomass reported earlier (Fig. 6; Hipkin *et al*., 2004) and suggested that growth of *Azolla* was not limited by nitrogen. Rhizobia are known epiphytes of cyanobacteria heterocysts (Stevenson & Waterbury, 2006). Possibly, the heterotrophic Rhizobiales help to lower the massive amounts of O_2_ released from leaf cell photosystem II activity at daytime in the leaf pockets, thereby preserving nitrogenase efficiency inside the heterocysts. Rhizobia may have adapted to survive the micro‐oxic environment they create, particularly at night, by respiring nitrate or nitrite. *Bradirhizobium japonicum* in soybean nodules is responsible for the bulk of N_2_O emissions when flooding soybeans: plants nodulated with *B. japonicum* mutants with a defect in NapA nitrate reductase producing nitrite emitted less N_2_O whilst plants with a defect in N_2_O reductase emitted more N_2_O (Tortosa *et al*., 2015). In *Azolla*, as in legumes, therefore, the denitrification pathway may present an adaptive advantage even though it may constitute futile cycling: survival of the bacteria when O_2_ levels are low. Direct N_2_O release from surface‐sterilized *Azolla* containing the Rhizobiales genomes could not be detected in this study, however, even under micro‐oxic conditions and after a prolonged night.

Possibly, endophyte communities co‐evolve with *Azolla*, and the metagenome is the unit that undergoes selection by the environment. This would be demonstrated if phylogenetic relationships of *Azolla* and its endophytes were to mirror one another, and if the endophytes were shown to be transmitted vertically upon sexual reproduction of *Azolla* by way of spores. Vertical transmission has been demonstrated for *N. azollae* in *A. filiculoides* (Ran *et al*., 2010), and it is entirely possible that the rhizobia reported here are similarly transmitted together with *N. azollae* in the megasporangiate sori of *A. filiculoides* (Carrapiço, 1991; Zheng *et al*., 2009). Phylogenetic studies are underway to verify this because, if true, it would imply that crop breeding approaches would have to consider endophytic communities.

### Nitrification: how could nitrate and nitrite be formed from the NH_4_^+^ released by *Nostoc azollae?*

Because bacterial endophytes from rice roots contained the AmoA (pfam 05145) ammonia monooxygenase (Sessitsch *et al*., 2012), which converts ammonium to nitrate, it is plausible that *Azolla* endophytes still awaiting characterization may be capable of converting the ammonium released by *N. azollae* to nitrate. Alternatively, many N_2_‐fixing plants are capable of phototrophic nitrification (Hipkin *et al*., 2004). In several leguminous plants, malonate is transformed via monoamide to 3‐nitropropionic acid (3‐NPA) and then to nitrate and nitrite (Francis *et al*., 2013). 3‐NPA is an inhibitor of mitochondrial succinate dehydrogenase (E.C. 1.3.5.1) and is therefore a strong antigrazing compound. It has been shown to accumulate at high levels in aquatic plants that fix N_2_ (e.g. *Lotus*), and is inactivated by the by 3‐NPA oxidases detected in a leguminous herb and characterized in *Pseudomonas aeruginosa*,* Burkholderia phytofirmans* and fungi (Nishino *et al*., 2010; Francis *et al*., 2013; Salvi *et al*., 2014). It will be important to decipher whether nitrification reactions occur within the leaf pocket or inside the fern cells. The combination of nitrifying and denitrifying endophytes could permit *Azolla* to cope with surplus levels of NH_4_^+^ from *N. azollae* or micro‐oxic ditch waters when phosphate availability is limiting and therefore contribute to defining the aquatic fern's ecological niche.

## Author contributions

L.W.D., P.B., H.B., A.M.B., G‐J.R. and H.S. designed and carried out the experiments involving the environmental samples, recruitment analyses, dinitrogen fixation analyses and detection of nitrous oxide release. H.S., B.H., N.K., A.W. and A.B. carried out long‐read sequencing and assembly of the *A. filiculoides* genome. F‐W.L., S.C., X.L., G.K‐S.W. and K.P. designed and carried out the experiments that provided the short sequence reads from the differing *Azolla* species. L.W.D., P.B., H.B., K.P. and H.S. wrote the manuscript, which was reviewed by all other authors.

## Supporting information

Please note: Wiley Blackwell are not responsible for the content or functionality of any Supporting Information supplied by the authors. Any queries (other than missing material) should be directed to the *New Phytologist* Central Office.

**Fig. S1** Relative abundance of classes in cultured *Azolla* species and *A. filiculoides* collected in the Dutch ditch.
**Fig. S2** Relative abundance of orders in cultured *Azolla* species and *A. filiculoides* collected in the Dutch ditch.
**Fig. S3** Recruitment frequencies on the Rhizobiales scaffolds comparing whole plant, enriched leaf pocket juice and surrounding water.
**Fig. S4** Full‐genome recruitment analyses.
**Fig. S5** Genome region surrounding the nitrite reductase from *Sinorhizobium*‐like and closely related bacteria.
**Fig. S6** Genome region surrounding the nitric oxide reductase from *Sinorhizobium*‐like and closely related bacteria.
**Fig. S7** Nitrous oxide reductase from *Agrobacterium‐*like is truncated.
**Fig. S8** Nitric oxide reductase B (large subunit) from *Agrobacterium‐*like.
**Fig. S9** ^15^N uptake by *A. filicuoides* with or without cyanobacteria after 24 h in ^15^N_2_‐enriched air.
**Table S1** Characteristics of sequencing data from environmental samples
**Table S2** Enzymes of the nitrogen metabolism in the *Rhizobiales* scaffolds

## References

[nph14843-bib-0001] Adams DG , Bergman B , Nierzwicki‐Bauer S , Duggan PS , Rai AN , Schüßler A . 2013 Cyanobacterial–plant symbioses In: Rosenberg E, DeLong EF, Lory S, Stackebrandt E, Fabiano T, eds. The prokaryotes. Berlin, Germany: Springer, 359–400.

[nph14843-bib-0002] Anglade J , Billen G , Garnier J . 2015 Relationships for estimating N_2_ fixation in legumes: incidence for N balance of legume‐based cropping systems in Europe. Ecosphere 6: 1–24.

[nph14843-bib-0003] Aziz RK , Bartels D , Best AA , DeJongh M , Disz T , Edwards RA , Formsma K , Gerdes S , Glass EM , Kubal M *et al* 2008 The RAST Server: rapid annotations using subsystems technology. BMC Genomics 9: 75. 18261238 10.1186/1471-2164-9-75 PMC2265698

[nph14843-bib-0004] Becerra M , Murgueitio E , Reyes G , Preston TR . 1990 *Azolla filiculoides* as partial replacement for traditional protein supplements in diets for growing‐fattening pigs based on sugar cane juice. Livestock Research for Rural Development 2: 15–22.

[nph14843-bib-0005] Bolger AM , Lohse M , Usadel B . 2014 Trimmomatic: a flexible trimmer for Illumina sequence data. Bioinformatics 30: 2114–2120. 24695404 10.1093/bioinformatics/btu170 PMC4103590

[nph14843-bib-0006] Braun‐Howland EB , Lindblad P , Nierzwicki‐Bauer SA , Bergman B . 1988 Dinitrogenase reductase (Fe‐protein) of nitrogenase in the cyanobacterial symbionts of three *Azolla* species: localization and sequence of appearance during heterocyst differentiation. Planta 176: 319–322. 24220860 10.1007/BF00395412

[nph14843-bib-0007] Brouwer P , Bräutigam A , Külahoglu C , Tazelaar AOE , Kurz S , Nierop KGJ , van der Werf A , Weber APM , Schluepmann H . 2014 *Azolla* domestication towards a biobased economy? New Phytologist 202: 1069–1082. 24494738 10.1111/nph.12708

[nph14843-bib-0600] Brouwer P , Bräutigam A , Buijs VA , Tazelaar AO , van der Werf A , Schlüter U , Reichart GJ , Bolger A , Usadel B , Weber AP , *et al* 2017 Metabolic adaptation, a specialized leaf organ structure and vascular responses to diurnal N_2_ fixation by *Nostoc azollae* sustain the astonishing productivity of *Azolla* ferns without nitrogen fertilizer. Frontiers in Plant Science 8: 442. 28408911 10.3389/fpls.2017.00442 PMC5374210

[nph14843-bib-0008] Cao HX , Schmutzer T , Scholz U , Pecinka A , Schubert I , Vu GT . 2015 Metatranscriptome analysis reveals host‐microbiome interactions in traps of carnivorous *Genlisea* species. Frontiers in Microbiology 6: 526 26236284 10.3389/fmicb.2015.00526 PMC4500957

[nph14843-bib-0009] Carr D , Lewin‐Koh N , Maechler M . 2015 R hexbin: hexagonal binning routines. [WWW document] URL https://cran.r-project.org/web/packages/hexbin/index.html [accessed 29 January 2016].

[nph14843-bib-0010] Carrapiço F . 1991 Are bacteria the third partner of the *Azolla–Anabaena* symbiosis? Plant and Soil 137: 157–160.

[nph14843-bib-0011] Carrapiço F . 2010 *Azolla* as a superorganism: its implication in symbiotic studies In: Seckbach J, Grube M, eds. Symbioses and stress: joint ventures in biology. Dordrecht, the Netherlands: Springer, 225–241.

[nph14843-bib-0012] Castelle CJ , Hug LA , Wrighton KC , Thomas BC , Williams KH , Wu D , Tringe SG , Singer SW , Eisen JA , Banfield JF . 2013 Extraordinary phylogenetic diversity and metabolic versatility in aquifer sediment. Nature Communications 4: 2120. 10.1038/ncomms3120 PMC3903129 23979677

[nph14843-bib-0013] Charuvaka A , Rangwala H . 2011 Evaluation of short read metagenomic assembly. BMC Genomics 12: S8. 10.1186/1471-2164-12-S2-S8 PMC3194239 21989307

[nph14843-bib-0014] Dowle M , Short T , Lianoglou S , Srinivasan A . 2014 R: data.table. CRAN. [WWW document] URL http://r-datatable.com [accessed 1 February 2017].

[nph14843-bib-0015] El‐Chakhtoura J , Prest E , Saikaly P , van Loosdrecht M , Hammes F , Vrouwenvelder H . 2015 Dynamics of bacterial communities before and after distribution in a full‐scale drinking water network. Water Research 74: 180–190. 25732558 10.1016/j.watres.2015.02.015

[nph14843-bib-0016] Forni C , Riov J , Caiola MG , Tel‐Or E . 1992 Indole‐3‐acetic acid (IAA) production by *Arthrobacter* species isolated from *Azolla* . Microbiology 138: 377–381. 10.1099/00221287-138-2-377 1564446

[nph14843-bib-0017] Forni C , Tel‐Or E , Bar E , Caiola MG . 1991 Effects of antibiotic treatments on *Azolla–Anabaena* and *Arthrobacter* In: Polsinelli M, Materassi R, Vincenzini M, eds. Developments in plant and soil sciences. Dordrecht, the Netherlands: Springer, 457–461.

[nph14843-bib-0018] Francis K , Smitherman C , Nishino SF , Spain JC , Gadda G . 2013 The biochemistry of the metabolic poison propionate 3‐nitronate and its conjugate acid, 3‐nitropropionate. IUBMB Life 65: 759–768. 23893873 10.1002/iub.1195

[nph14843-bib-0019] Gebhardt JS , Nierzwicki‐Bauer SA . 1991 Identification of a common cyanobacterial symbiont associated with *Azolla* spp. through molecular and morphological characterization of free‐living and symbiotic cyanobacteria. Applied and Environmental Microbiology 57: 2141–2146. 1685078 10.1128/aem.57.8.2141-2146.1991 PMC183541

[nph14843-bib-0020] Hill MP . 1998 Life history and laboratory host range of *Stenopelmus rufinasus*, a natural enemy for *Azolla filiculoides* in South Africa. BioControl 43: 215–224.

[nph14843-bib-0021] Hipkin CR , Simpson DJ , Wainwright SJ , Salem MA . 2004 Nitrification by plants that also fix nitrogen. Nature 430: 98–101. 15229604 10.1038/nature02635

[nph14843-bib-0022] Horton MW , Bodenhausen N , Beilsmith K , Meng D , Muegge BD , Subramanian S , Vetter MM , Vilhjalmsson BJ , Nordborg M , Gordon JI *et al* 2014 Genome‐wide association study of *Arabidopsis thaliana* leaf microbial community. Nature Communications 5: 5320. 10.1038/ncomms6320 PMC4232226 25382143

[nph14843-bib-0023] Jensen ES , Peoples MB , Boddey RM , Gresshoff PM , Henrik HN , Alves BJR , Morrison MJ . 2012 Legumes for mitigation of climate change and the provision of feedstock for biofuels and biorefineries. A review. Agronomy for Sustainable Development 32: 329–364.

[nph14843-bib-0024] Kanehisa M , Goto S , Furumichi M , Tanabe M , Hirakawa M . 2010 KEGG for representation and analysis of molecular networks involving diseases and drugs. Nucleic Acids Research 38: D355–D360. 19880382 10.1093/nar/gkp896 PMC2808910

[nph14843-bib-0025] Koren S , Schatz MC , Walenz BP , Martin J , Howard JT , Ganapathy G , Wang Z , Rasko DA , McCombie WR , Jarvis ED *et al* 2012 Hybrid error correction and *de novo* assembly of single‐molecule sequencing reads. Nature Biotechnology 30: 693–700. 10.1038/nbt.2280 PMC3707490 22750884

[nph14843-bib-0026] Lagesen K , Hallin P , Rødland EA , Staerfeldt H‐H , Rognes T , Ussery DW . 2007 RNAmmer: consistent and rapid annotation of ribosomal RNA genes. Nucleic Acids Research 35: 3100–3108. 17452365 10.1093/nar/gkm160 PMC1888812

[nph14843-bib-0027] Langmead B , Salzberg SL . 2012 Fast gapped‐read alignment with Bowtie 2. Nature Methods 9: 357–359. 22388286 10.1038/nmeth.1923 PMC3322381

[nph14843-bib-0028] Leonard MT , Davis‐Richardson AG , Ardissone AN , Kemppainen KM , Drew JC , Ilonen J , Knip M , Simell O , Toppari J , Veijola R *et al* 2014 The methylome of the gut microbiome: disparate Dam methylation patterns in intestinal *Bacteroides dorei* . Frontiers in Microbiology 5: 1–6. 25101067 10.3389/fmicb.2014.00361 PMC4101878

[nph14843-bib-0029] Lindblad P , Bergman B , Nierzwicki‐Bauer SA . 1991 Immunocytochemical localization of nitrogenase in bacteria symbiotically associated with *Azolla* spp. Applied and Environmental Microbiology 57: 3637–3640. 1785936 10.1128/aem.57.12.3637-3640.1991 PMC184025

[nph14843-bib-0031] Lundberg DS , Lebeis SL , Paredes SH , Yourstone S , Gehring J , Malfatti S , Tremblay J , Engelbrektson A , Kunin V , Del Rio TG *et al* 2012 Defining the core *Arabidopsis thaliana* root microbiome. Nature 488: 86–90. 22859206 10.1038/nature11237 PMC4074413

[nph14843-bib-0032] Lutz KA , Wang W , Zdepski A , Michael TP . 2011 Isolation and analysis of high quality nuclear DNA with reduced organellar DNA for plant genome sequencing and resequencing. BMC Biotechnology 11: 54. 21599914 10.1186/1472-6750-11-54 PMC3131251

[nph14843-bib-0033] Miller CS , Baker BJ , Thomas BC , Singer SW , Banfield JF . 2011 EMIRGE: reconstruction of full‐length ribosomal genes from microbial community short read sequencing data. Genome Biology 12: R44. 21595876 10.1186/gb-2011-12-5-r44 PMC3219967

[nph14843-bib-0034] Mizuno CM , Rodriguez‐Valera F , Kimes NE , Ghai R . 2013 Expanding the marine virosphere using metagenomics. PLoS Genetics 9: e1003987. 24348267 10.1371/journal.pgen.1003987 PMC3861242

[nph14843-bib-0035] Myers EW , Sutton GG , Delcher AL , Dew IM , Fasulo DP , Flanigan MJ , Kravitz SA , Mobarry CM , Reinert KH , Remington KA *et al* 2000 A whole‐genome assembly of *Drosophila* . Science 287: 2196–2204. 10731133 10.1126/science.287.5461.2196

[nph14843-bib-0609] Nagalingum NS , Schneider H , Pryer KM . 2006 Comparative morphology of reproductive structures in heterosporous water ferns and a reevaluation of the sporocarp. International Journal of Plant Sciences 167: 805–815.

[nph14843-bib-0036] Nishino SF , Shin KA , Payne RB , Spain JC . 2010 Growth of bacteria on 3‐nitropropionic acid as a sole source of carbon, nitrogen, and energy. Applied and Environmental Microbiology 76: 3590–3598. 20382807 10.1128/AEM.00267-10 PMC2876434

[nph14843-bib-0037] Orr J , Haselkorn R . 1982 Regulation of glutamine synthetase activity and synthesis in free‐living and symbiotic *Anabaena* spp. Journal of Bacteriology 152: 626–635. 6127334 10.1128/jb.152.2.626-635.1982 PMC221509

[nph14843-bib-0038] Overbeek R , Olson R , Pusch GD , Olsen GJ , Davis JJ , Disz T , Edwards RA , Gerdes S , Parrello B , Shukla M *et al* 2014 The SEED and the Rapid Annotation of microbial genomes using Subsystems Technology (RAST). Nucleic Acids Research 42: D206–D214. 24293654 10.1093/nar/gkt1226 PMC3965101

[nph14843-bib-0039] Pašić L , Rodriguez‐Mueller B , Martin‐Cuadrado AB , Mira A , Rohwer F , Rodriguez‐Valera F . 2009 Metagenomic islands of hyperhalophiles: the case of *Salinibacter ruber* . BMC Genomics 10: 570. 19951421 10.1186/1471-2164-10-570 PMC2800850

[nph14843-bib-0040] Peñuelas J , Terradas J . 2014 The foliar microbiome. Trends in Plant Science 19: 278–280. 24439491 10.1016/j.tplants.2013.12.007

[nph14843-bib-0041] Pereira AL , Vasconcelos V . 2014 Classification and phylogeny of the cyanobiont *Anabaena azollae* Strasburger: an answered question? International Journal of Systematic and Evolutionary Microbiology 64: 1830–1840. 24737795 10.1099/ijs.0.059238-0

[nph14843-bib-0042] Perkins SK , Peters GA . 1993 The *Azolla* and *Anabaena* symbiosis: endophyte continuity in the *Azolla* life cycle is facilitated by epidermal trichomes. New Phytologist 123: 53–64.

[nph14843-bib-0043] Plazinski J , Taylor R , Shaw W , Croft L , Rolfe BG , Gunning BE . 1990 Isolation of *Agrobacterium* sp., strain from the *Azolla* leaf cavity. FEMS Microbiology Letters 70: 55–59.

[nph14843-bib-0044] Quast C , Pruesse E , Yilmaz P , Gerken J , Schweer T , Yarza P , Peplies J , Glöckner FO . 2013 The SILVA ribosomal RNA gene database project: improved data processing and web‐based tools. Nucleic Acids Research 41: D590–D596. 23193283 10.1093/nar/gks1219 PMC3531112

[nph14843-bib-0045] R Core Team . 2013 R: a language and environment for statistical computing. Vienna, Austria: R Foundation for Statistical Computing.

[nph14843-bib-0046] Ran L , Larsson J , Vigil‐Stenman T , Nylander JAA , Ininbergs K , Zheng W‐W , Lapidus A , Lowry S , Haselkorn R , Bergman B . 2010 Genome erosion in a nitrogen‐fixing vertically transmitted endosymbiotic multicellular cyanobacterium. PLoS ONE 5: e11486. 20628610 10.1371/journal.pone.0011486 PMC2900214

[nph14843-bib-0047] Salvi F , Agniswamy J , Yuan H , Vercammen K , Pelicaen R , Cornelis P , Spain JC , Weber IT , Gadda G . 2014 The combined structural and kinetic characterization of a bacterial nitronate monooxygenase from *Pseudomonas aeruginosa* PAO1 establishes NMO class I and II. Journal of Biological Chemistry 289: 23764–23775. 25002579 10.1074/jbc.M114.577791 PMC4156069

[nph14843-bib-0048] Schloss PD , Westcott SL , Ryabin T , Hall JR , Hartmann M , Hollister EB , Lesniewski RA , Oakley BB , Parks DH , Robinson DJ *et al* 2009 Introducing Mothur: open‐source, platform‐independent, community‐supported software for describing and comparing microbial communities. Applied and Environmental Microbiology 75: 7537–75341. 19801464 10.1128/AEM.01541-09 PMC2786419

[nph14843-bib-0049] Sessitsch A , Hardoim P , Döring J , Weilharter A , Krause A , Woyke T , Mittler B , Hauberg‐Lotte L , Friedrich F , Rahalker M *et al* 2012 Functional characteristics of an endophyte community colonizing rice roots as revealed by metagenomic analysis. Molecular Plant–Microbe Interactions 25: 28–36. 21970692 10.1094/MPMI-08-11-0204

[nph14843-bib-0050] Simonsen AK , Stinchcombe JR . 2014 Herbivory eliminates fitness costs of mutualism exploiters. New Phytologist 202: 651–661. 24428169 10.1111/nph.12668

[nph14843-bib-0051] Stevenson BS , Waterbury JB . 2006 Isolation and identification of an epibiotic bacterium associated with heterocystous *Anabaena* cells. Biological Bulletin 210: 73–77. 16641512 10.2307/4134596

[nph14843-bib-0052] Stone JK , Bacon CW , White JF . 2000 An overview of endophytic microbes: endophytism defined In: Bacon CW, White JF, eds. Microbial endophytes. New York, NY, USA: Marcel Dekker, 3–29.

[nph14843-bib-0053] Tange O . 2011 GNU Parallel: the command‐line power tool. USENIX Magazine 36: 42–47.

[nph14843-bib-0054] Tortosa G , Hidalgo A , Salas A , Bedmar EJ , Mesa S , Delgado MJ . 2015 Nitrate and flooding induce N_2_O emissions from soybean nodules. Symbiosis 67: 125–133.

[nph14843-bib-0055] Turner TR , James EK , Poole PS . 2013 The plant microbiome. Genome Biology 14: 1–10. 10.1186/gb-2013-14-6-209 PMC3706808 23805896

[nph14843-bib-0056] Tyson GW , Chapman J , Hugenholtz P , Allen EE , Ram RJ , Richardson PM , Solovyev VV , Rubin EM , Rokhsar DS , Banfield JF . 2004 Community structure and metabolism through reconstruction of microbial genomes from the environment. Nature 428: 37–43. 14961025 10.1038/nature02340

[nph14843-bib-0057] Vance CP . 2002 Root–bacteria interactions: symbiotic N_2_ fixation In: Waisel Y, Eshel Y, Kafkafi U, eds. Plant roots: the hidden half, *3^rd^ edn* New York, NY, USA: Marcel Dekker, 839–868.

[nph14843-bib-0058] Vigil‐Stenman T , Larsson J , Nylander JA , Bergman B . 2015 Local hopping mobile DNA implicated in pseudogene formation and reductive evolution in an obligate cyanobacteria–plant symbiosis. BMC Genomics 16: 193. 25885210 10.1186/s12864-015-1386-7 PMC4369082

[nph14843-bib-0059] Watanabe I . 1992 Biofertilizer germplasm collections at IRRI. Los Banos, the Philippines: International Rice Research Institute.

[nph14843-bib-0060] Wickham H . 2011 Ggplot2. Wiley Interdisciplinary Reviews Computational Statistics 3: 180–185.

[nph14843-bib-0061] Wrighton KC , Castelle CJ , Wilkins MJ , Hug LA , Sharon I , Thomas BC , Mullin SW , Nicora CD , Singh A , Lipton MS *et al* 2014 Metabolic interdependencies between phylogenetically novel fermenters and respiratory organisms in an unconfined aquifer. ISME Journal 8: 1452–1463. 24621521 10.1038/ismej.2013.249 PMC4069391

[nph14843-bib-0062] Xie C , Goi CL , Huson DH , Little PF , Williams RB . 2016 RiboTagger: fast and unbiased 16S/18S profiling using whole community shotgun metagenomic or metatranscriptome surveys. BMC Bioinformatics 17: 277. 28155666 10.1186/s12859-016-1378-x PMC5259810

[nph14843-bib-0030] Zependa Mendoza ML , Sicheritz‐Pontén T , Gilbert MTP . 2015 Environmental genes and genomes: understanding the differences and challenges in the approaches and software for their analyses. Briefings in Bioinformatics 16: 745–758. 25673291 10.1093/bib/bbv001 PMC4570204

[nph14843-bib-0063] Zheng W , Bergman B , Chen B , Zheng S , Xiang G , Rasmussen U . 2009 Cellular responses in the cyanobacterial symbiont during its vertical transfer between plant generations in the *Azolla microphylla* symbiosis. New Phytologist 181: 53–61. 19076717 10.1111/j.1469-8137.2008.02644.x

