# Supplementary material for: Is there foul play in the leaf pocket? The metagenome of floating fern Azolla reveals endophytes that do not fix N2 but may denitrify
Source: New Phytol. 2017 Oct 30;217(1):453–66. doi: 10.1111/nph.14843 (PMC5901025; doi:10.1111/nph.14843)
Supplement: Supplementary file 1 — Fig. S1 Relative abundance of classes in cultured Azolla species and A. filiculoides collected in the Dutch ditch. Fig. S2 Relative abundance of orders in cultured Azolla species and A. filiculoides collected in the Dutch ditch. Fig. S3 Recruitment frequencies on the Rhizobiales scaffolds comparing whole plant, enriched leaf pocket juice and surrounding water. Fig. S4 Full‐genome recruitment analyses. Fig. S5 Genome region surrounding the nitrite reductase from Sinorhizobium‐like and closely related bacteria. Fig. S6 Genome region surrounding the nitric oxide reductase from Sinorhizobium‐like and closely related bacteria. Fig. S7 Nitrous oxide reductase from Agrobacterium‐like is truncated. Fig. S8 Nitric oxide reductase B (large subunit) from Agrobacterium‐like. Fig. S9 15N uptake by A. filicuoides with or without cyanobacteria after 24 h in 15N2‐enriched air. Table S1 Characteristics of sequencing data from environmental samples Table S2 Enzymes of the nitrogen metabolism in the Rhizobiales scaffolds [file NPH-217-453-s001.pdf]

***New Phytologist* Supporting Information**

Article title:

Is there foul play in the pocket? The metagenome of floating fern *Azolla* reveals endophytes that do not fix N_2 but may denitrify.

Authors:

Laura W Dijkhuizen, Paul Brouwer, Henk Bolhuis, Gert-Jan Reichart, Nils Koppers, Bruno Huettel, Anthony M Bolger, Fay-Wei Li, Shifeng Cheng, Xin Liu, Gane Ka-Shu Wong, Kathleen Pryer, Andreas Weber, Andrea Bräutigam and Henriette Schluepmann.

Article acceptance date: 05 September 2017

The following Supporting Information is available for this article:

Table S1 Characteristics of sequencing data from environmental samples.

Table S2 Enzymes of the nitrogen metabolism in the *Rhizobiales* scaffolds.

Fig. S1 Relative abundance of classes in cultured *Azolla* species and *A. filiculoides* collected in the Dutch ditch.

Fig. S2 Relative abundance of orders in cultured *Azolla* species and *A. filiculoides* collected in the Dutch ditch.

Fig. S3 Recruitment frequencies on the Rhizobiales scaffolds comparing whole plant, enriched leaf pocket juice and surrounding water.

Fig. S4 Full-genome recruitment analyses.

Fig. S5 Genome region surrounding the nitrite reductase from *Sinorhizobium*-like and closely related bacteria.

Fig. S6 Genome region surrounding the nitric oxide reductase for *Sinorhizobium*-like and closely related bacteria.

Fig. S7 Nitrous oxide reductase from *Agrobacterium*-like is truncated.

Fig. S8 Nitric oxide reductase B (large subunit) from *Agrobacterium*-like.

Fig. S9 ^{15}N uptake by *A.filicuoides* with or without cyanobacteria after 24 h in $^{15}N_2$ enriched Air.

Table S1 Characteristics of sequencing data from environmental samples.

Sample name[1]	Raw reads (M)	Paired reads postQC (M)	Mean phred score paired reads postQC	Mean length paired reads post QC
P1	10.17	9.80	33.37	146.2
P2	11.10	10.73	33.35	146.28
P4	8.42	8.13	33.34	146.21
L1	10.94	10.61	33.41	147.4
L2	9.39	9.10	33.51	147.58
L3	10.15	9.77	33.49	147.56
W1	11.99	11.60	33.42	147.62
W2	9.88	9.57	33.37	147.54
W3	10.22	9.91	33.42	147.69

[1] DNA was extracted in three biological replicates from washed whole ferns (P), from leaf juice (L) or from the filtrate of the surrounding water (W).

Table S2 Enzymes of nitrogen metabolism in the *Sinorhizobium*-like and *Agrobacterium*-like scaffolds compared to those from the related rhizobia *Sinorhizobium meliloti* and *Rhizobium leguminosarum*[1].

KEGG map	Distinct EC	*Sinorhizobium*-like	*Agrobacterium*-like	*Sinorhizobium meliloti* 1021	*Rhizobium leguminosarum* bv. viciae 3841
Alanine, aspartate and glutamate metabolism	43	16 (37.2 %)	19 (44.2 %)	20 (46.5 %)	18 (41.9 %)
Arginine and proline metabolism	97	29 (29.9 %)	40 (41.2 %)	38 (39.2 %)	39 (40.2 %)
Glyoxylate and dicarboxylate metabolism	58	17 (29.3 %)	21 (36.2 %)	23 (39.7 %)	22 (37.9 %)
Methane metabolism	33	10 (30.3 %)	9 (27.3 %)	12 (36.4 %)	10 (30.3 %)
Nitrogen metabolism	57	16 (28.1 %)	16 (28.1 %)	19 (33.3 %)	15 (26.3 %)

[1] Generated by http://rast.nmpdr.org/seedviewer.cgi using the KEGG pathway comparison tool (Overbeek *et al.*, 2014). % relates the number of EC in each organism to the total number of distinct EC in each map.

Fig. S1 Relative abundance of classes in cultured *Azolla* species and *A. filiculoides* collected in the Dutch ditch. Sequencing data from cultured ferns originated from cultured *Azolla* species of which one *A. filiculoides* was rid of *N. azollae* (*A. filiculoides*-Sterilized; see also Table 1). Environmental sequencing data originated from enriched leaf-pocket content (L) whole plants (P) and surrounding water (W). All sequencing was processed as subsets of 10 M reads and 30 M reads. Taxonomy was assigned to rRNA fragments found in single reads by RiboTagger and to EMIRGE assembled rRNA genes by Mothur. i, refers to the average length of the DNA from the sequencing library.

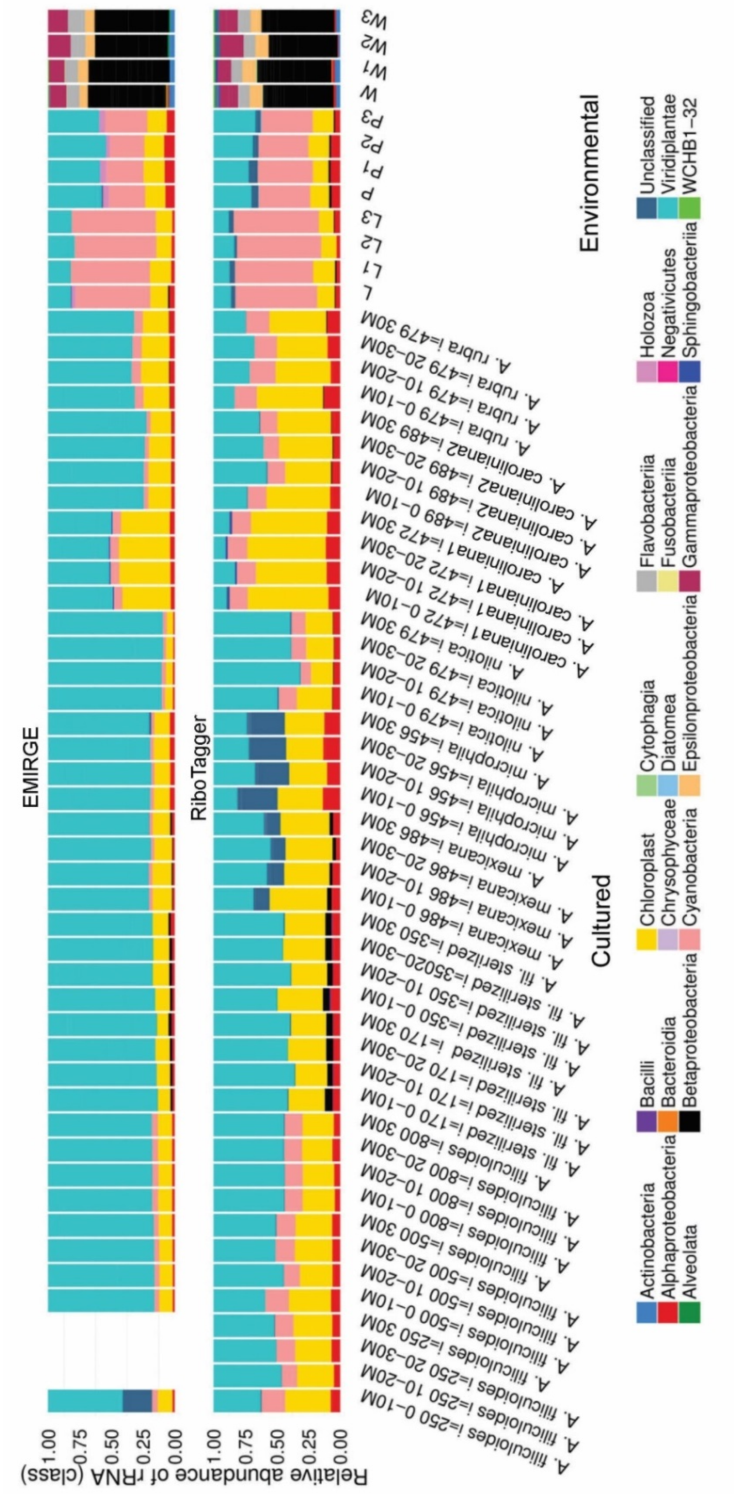

Fig. S2 Relative abundance of orders in cultured *Azolla* species and *A. filiculoides* collected in the Dutch ditch. Taxonomy was assigned to rRNA fragments found in single reads by RiboTagger and to rRNA genes assembled with EMIRGE by Mothur. Unclassified reads or Orders originating from fern nucleus, fern plastids and cyanobacteria are not shown. Environmental sequencing data originated from enriched leaf-pocket contents (L) whole plants (P) and surrounding water (W). Sequencing data of cultured *Azolla* originated from the species described in Table 1 of which one *A. filiculoides* was rid of *N. azollae* (*A. filiculoides*-Sterilized). All sequencing was processed as subsets of 10 M reads and 30 M reads.

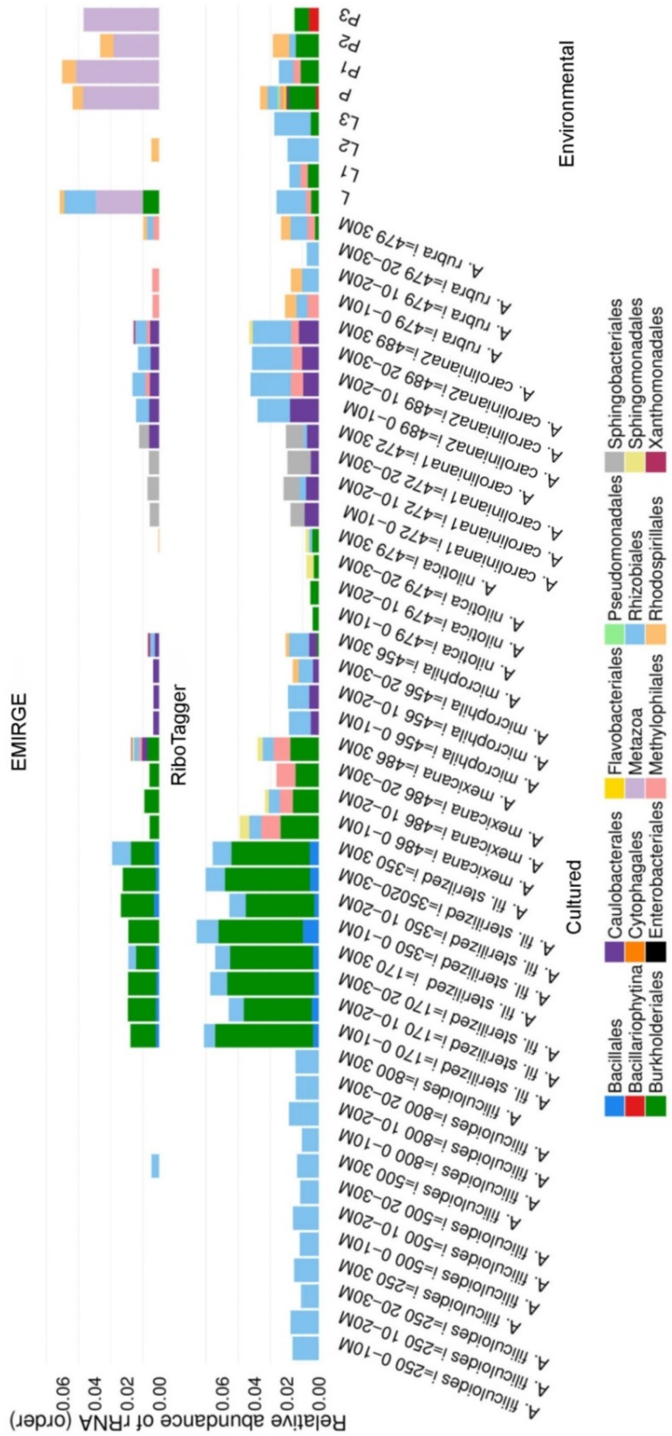

Fig. S3 Recruitment frequencies on the Rhizobiales scaffolds comparing whole plant, enriched leaf pocket juice and surrounding water. Short reads from environmental samples were recruited onto the 3.22 Mb Rhizobium and 4.96 Mb Shinella scaffolds from Celera assembly of *Azolla* genomic DNA. Short reads were either from samples of whole plant (P), enriched leaf pocket contents (L) or surrounding water (W). The data are averages from three independent replicates with standard deviations.

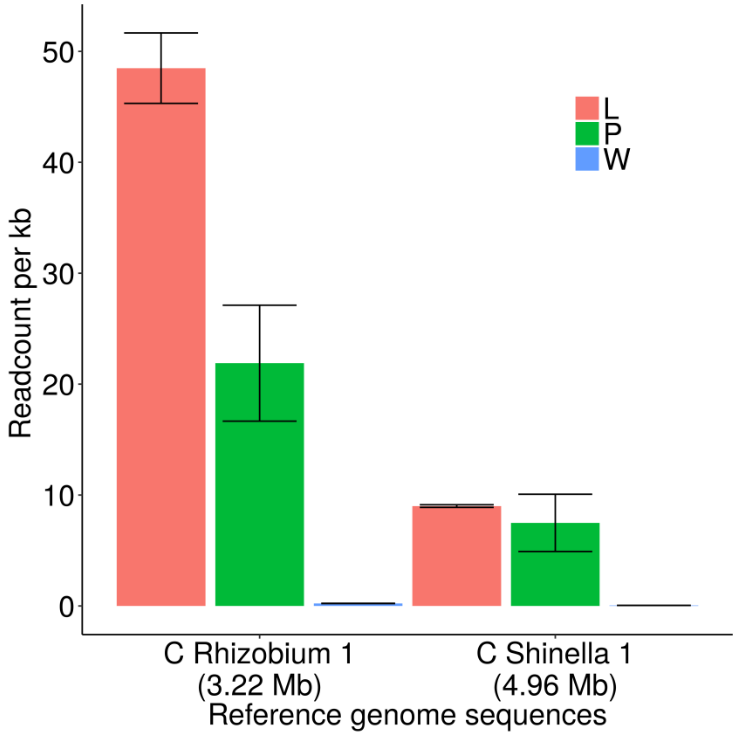

Fig. S4 Full-genome recruitment analyses. Scaffolds from assemblies of *Azolla* genomic DNA included the *Hydrocarboniphaga sp.* and the *Sinorhizobium*-like scaffolds. Reference genomes were as in Fig. 4 with in addition two accessions of *Sinorhizobium meliloti* (AL591688.1 and AKZZ01000000). Short reads used for the recruitment were from cultured ferns (Table 1) or from environmental samples collected in the ditch, whole plant (P), enriched leaf pocket contents (L) and surrounding water (W) as in Figs. 1-3.

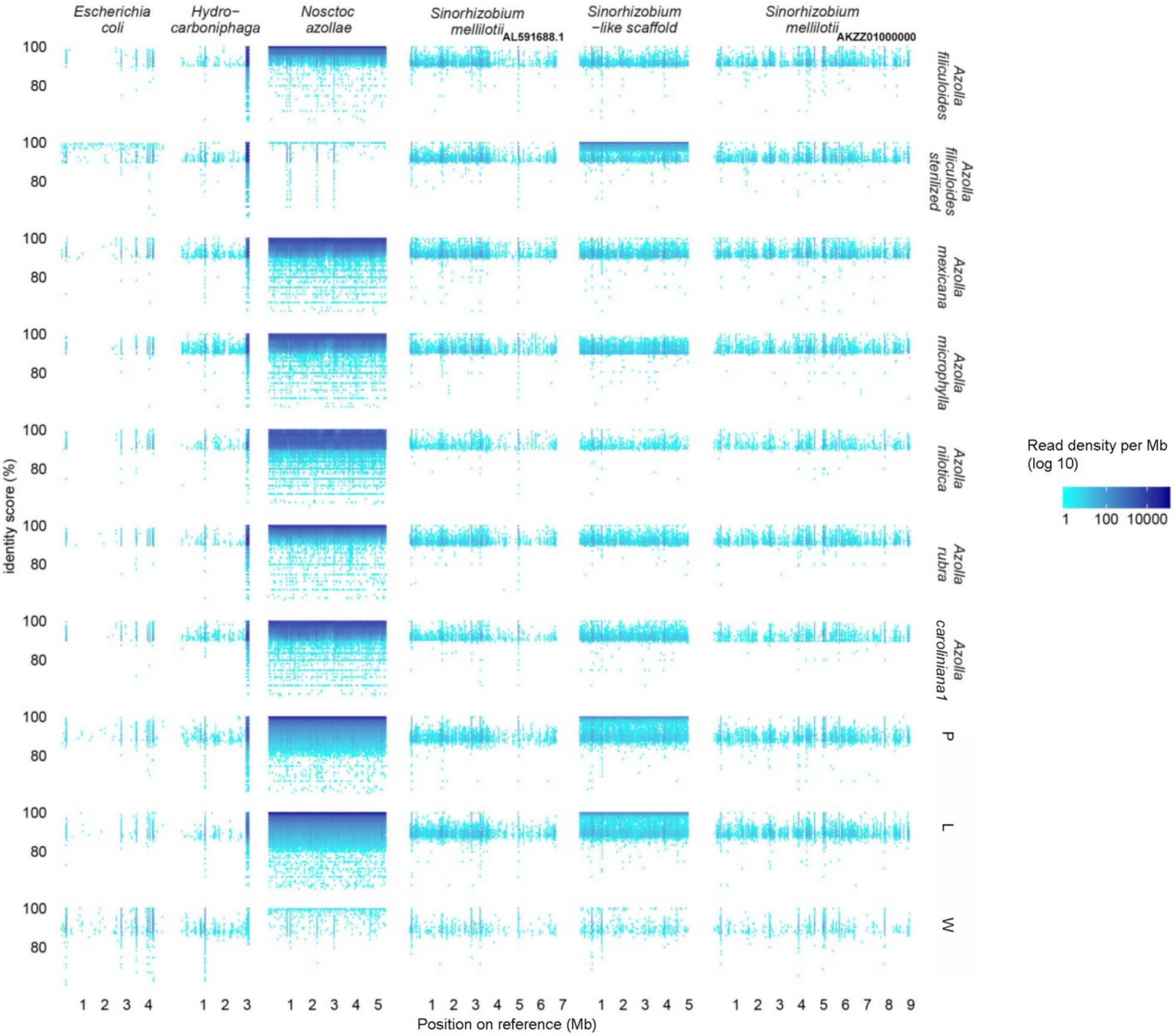

Fig. S5 Genome region surrounding the nitrite reductase from *Sinorhizobium*-like and closely related bacteria. Predicted annotation by RAST (Overbeek *et al.*, 2014). 1, Copper-containing nitrite reductase NirK (EC 1.7.2.1); 2, Nitrite reductase accessory protein NirV; 3, Nitric oxide -responding transcriptional regulator NnrR (Crp/Fnr family); 4, hypothetical protein; 5, NnrS protein involved in response to NO; 6, PaaD-like protein (DUF59) involved in Fe-S cluster assembly; 7, protein; 8, Nitric oxide reductase activation protein NorD.

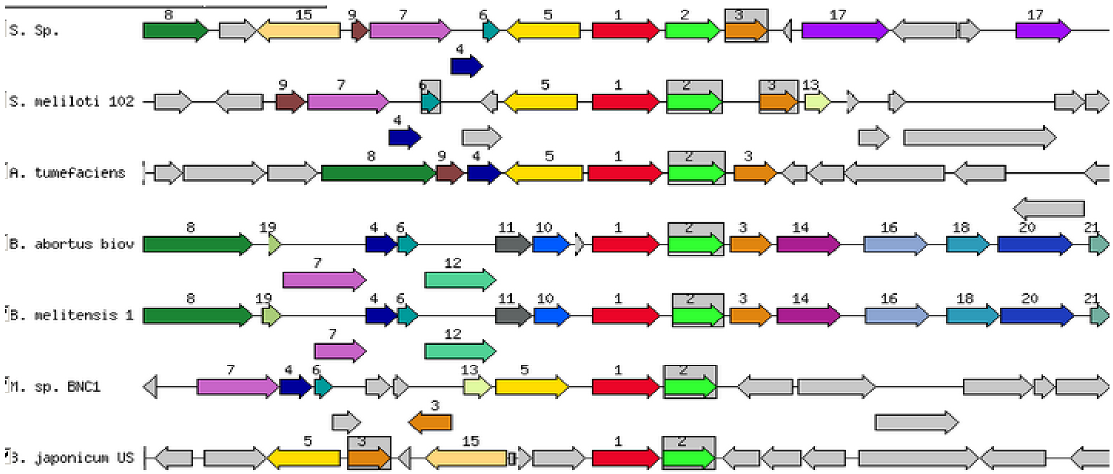

Fig. S6 Genome region surrounding the nitric oxide reductase for *Sinorhizobium*-like and closely related bacteria. 1, Nitric-oxide reductase subunit NorC (EC 1.7.99.7); 2, Nitric-oxide reductase subunit NorB (EC 1.7.99.7); 3, Nitric oxide reductase activation protein NorQ; 4, Nitric oxide reductase activation protein NorD; 5, Nitric oxide reductase activation protein NorE; 6, NnrS protein involved in response to NO; 7, NnrU family protein, required for expression of nitric oxide and nitrite reductases (Nir and Nor).

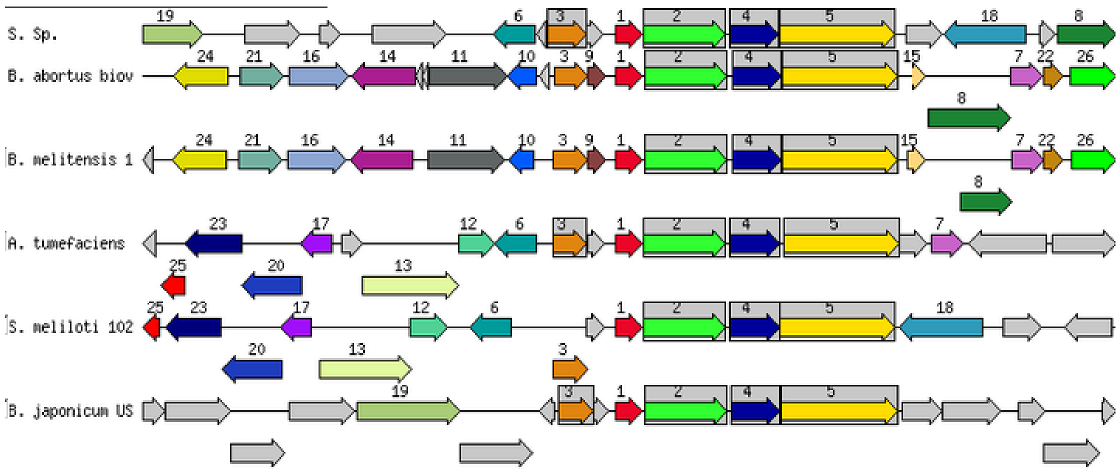

Fig. S7 Nitrous oxide reductase from *Agrobacterium*-like is truncated, it contains only the C-domain with the full CCD domain. 1, Nitrous-oxide reductase (EC 1.7.99.6 with left from it two transposase proteins in grey); 2, Nitrous oxide reductase maturation protein NosD; 3, Nitrous oxide reductase maturation protein NosF (ATPase); 4, Nitrous oxide reductase maturation transmembrane protein NosY; 5, Nitrous oxide reductase maturation protein, outer-membrane lipoprotein NosL; 6, Nitrous oxide reductase maturation protein NosR; 7, Nitrous oxide reductase maturation protein, outer-membrane lipoprotein NosL; 10, Nitric oxide reductase activation protein NorE; 11, NnrU family protein, required for expression of nitric oxide and nitrite reductases (Nir and Nor).

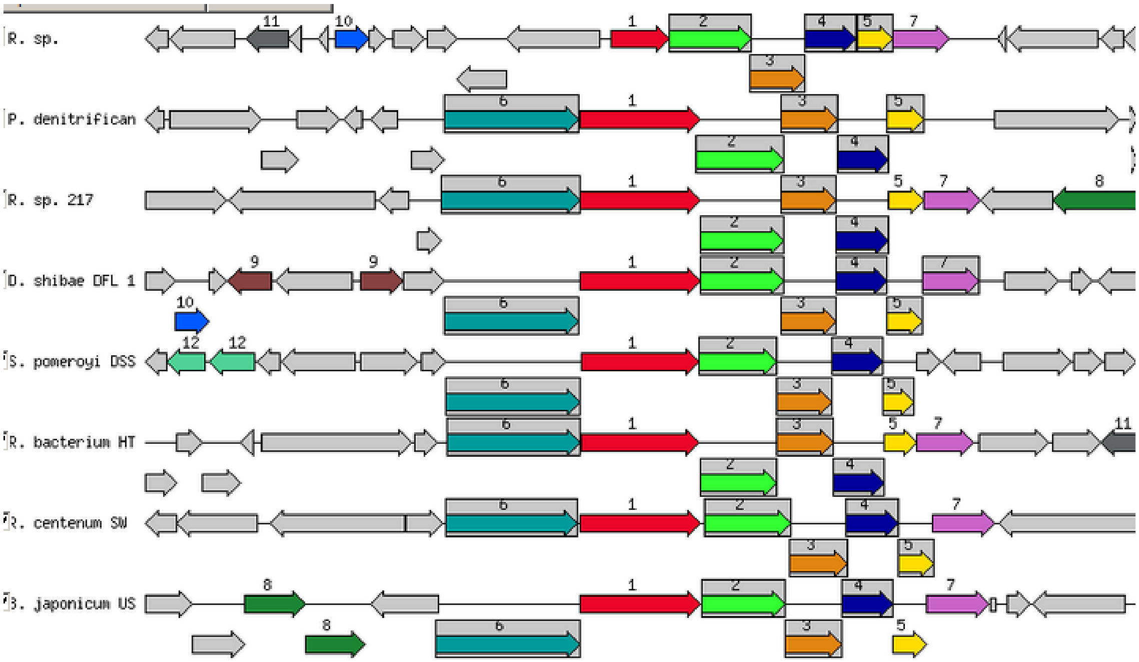

Fig. S8 Nitric oxide reductase B (large subunit) from *Agrobacterium*-like with insertion of the mobile element.1, Nitric-oxide reductase subunit B (EC 1.7.99.7) contains the entire conserved domain but to its right two mobile element proteins, the the nitrous oxide reductase (grey boxes); 2, Nitric-oxide reductase subunit C (EC 1.7.99.7); 5, Nitric oxide reductase activation protein NorE; 6, NnrS protein involved in response to NO; 7, NnrU family protein, required for expression of nitric oxide and nitrite reductases (Nir and Nor).

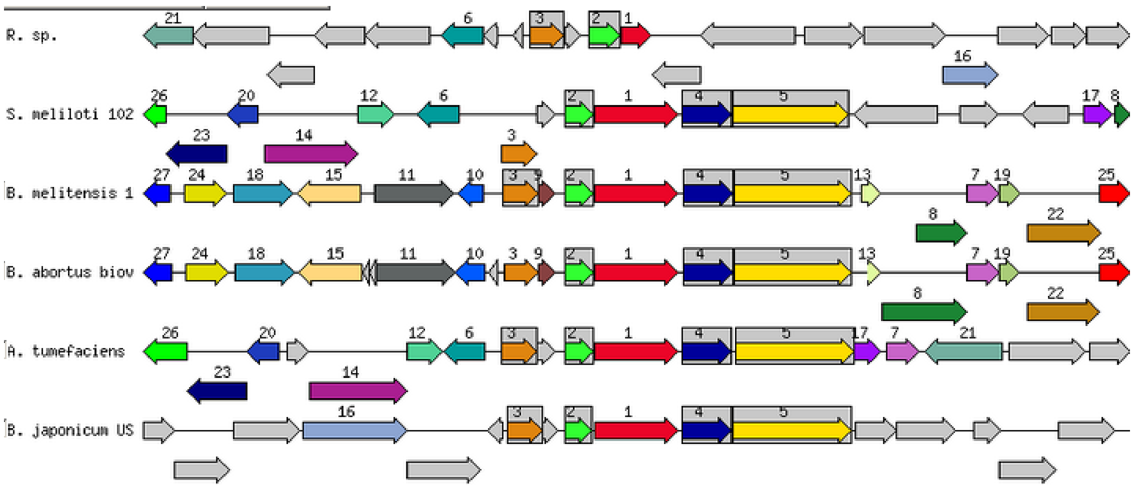

Fig. S9 ^{15}N uptake by *A. filicuoides* with and without cyanobacteria after 24 h in $^{15}N_2$ enriched Air. The ferns (100 mg FW) on IRRI-medium with 2 mM KNO_3 were incubated in $^{15}N_2$ enriched Air during one diel cycle (24 h), then snap frozen and freeze dried before ^{15}N and ^{14}N isotope determinations. Wild type ferns (+Cyano+N) and *A. filiculoides*-Sterilized without cyanobacteria but with the Rhizobiales (-Cyano+N) were compared to *A. filiculoides* -Sterilized boiled for 5 min before incubation (Boiled-Cyano+N). Averages from biological triplicates are shown with standard deviation.

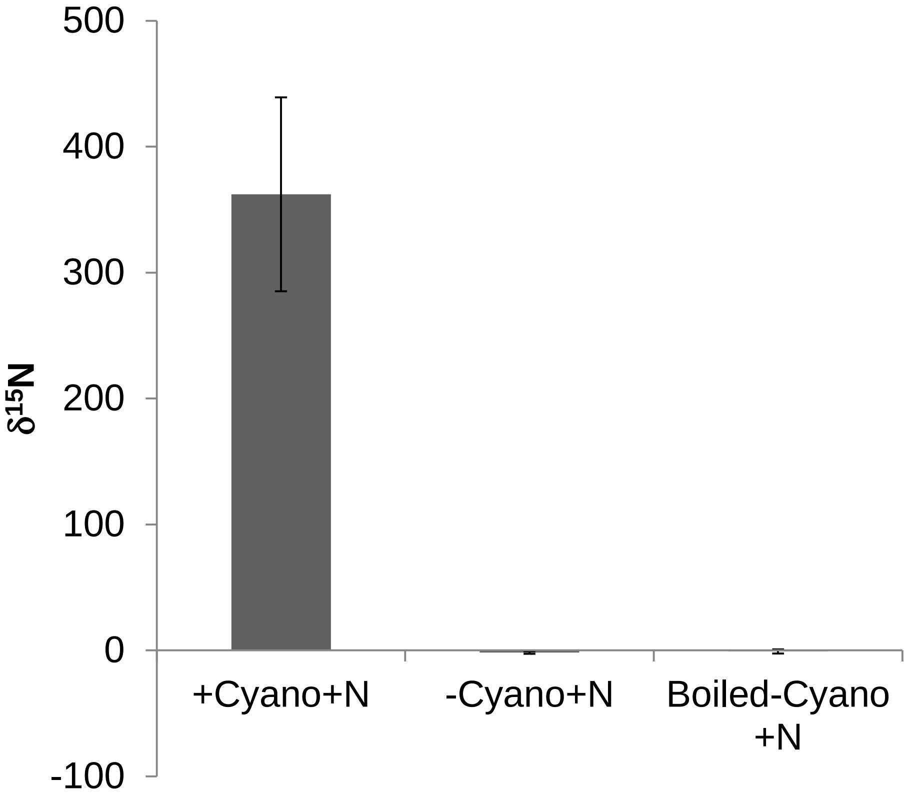

References

Overbeek R, Olson R, Pusch GD, Olsen GJ, Davis JJ, Disz T, Edwards RA, Gerdes S, Parrello B, Shukla M, *et al.* 2014. The SEED and the Rapid Annotation of microbial genomes using Subsystems Technology (RAST). *Nucleic Acids Research* **42**: D206-14.
